# Discovery of Novel Myristic Acid Derivatives as N-Myristoyltransferase Inhibitors: Design, Synthesis, Analysis, Computational Studies and Antifungal Activity

**DOI:** 10.3390/antibiotics12071167

**Published:** 2023-07-09

**Authors:** Saleem Javid, Hissana Ather, Umme Hani, Ayesha Siddiqua, Shaik Mohammad Asif Ansari, Dhivya Shanmugarajan, Honnavalli Yogish Kumar, Rajaguru Arivuselvam, Madhusudan N. Purohit, B. R. Prashantha Kumar

**Affiliations:** 1 Department of Pharmaceutical Chemistry, Farooqia College of Pharmacy, Mysore 570 015, Karnataka, India; stargm012@gmail.com; 2 Department of Pharmaceutical Chemistry, JSS College of Pharmacy, Mysore, JSS Academy of Higher Education & Research, Mysore 570 015, Karnataka, India; dskeerthanasai@gmail.com (D.S.); hyogishkumar@jssuni.edu.in (H.Y.K.); madhusudhanpurohit@jssuni.edu.in (M.N.P.); 3 Department of Pharmaceutical Chemistry, College of Pharmacy, King Khalid University, Abha 62529, Saudi Arabia; hissana@kku.edu.sa; 4 Department of Pharmaceutics, College of Pharmacy, King Khalid University, Abha 62529, Saudi Arabia; ummehaniahmed@gmail.com; 5 Department of Clinical Pharmacy, College of Pharmacy, King Khalid University, Abha 62529, Saudi Arabia; ayshafiq@kku.edu.sa (A.S.); aashaik@kku.edu.sa (S.M.A.A.); 6 Department of Pharmaceutical Biotechnology, JSS College of Pharmacy, Mysore, JSS Academy of Higher Education & Research, Mysore 570 015, Karnataka, India; arajaguru@jssuni.edu.in

**Keywords:** N-Myristoyltransferase inhibitors, antifungal activity, myristic acid derivatives, molecular docking, ADMET studies, NMT inhibition assay

## Abstract

In recent years, N-Myristoyltransferase (NMT) has been identified as a new target for the treatment of fungal infections. It is observed that at present, there are increased rates of morbidity and mortality due to fungal infections. Hence, a series of novel myristic acid derivatives were designed via molecular docking studies and ADMET studies by targeting NMT (N-Myristoyltransferase). The designed myristic acid derivatives were synthesized by converting myristic acid into myristoyl chloride and coupling it with aryl amines to yield corresponding myristic acid derivatives. The compounds were purified and characterized via FTIR, NMR and HRMS spectral analyses. In this study, we carried out a target NMT inhibition assay. In the NMT screening assay results, the compounds **3u, 3m** and **3t** showed better inhibition compared to the other myristic acid derivatives. In an in vitro antifungal evaluation, the myristic acid derivatives were assessed against *Candida albicans* and *Aspergillus niger* strains by determining their minimal inhibitory concentrations (MIC_50_). The compounds **3u, 3k**, **3r** and **3t** displayed superior antifungal capabilities against *Candida albicans*, and the compounds **3u, 3m** and **3r** displayed superior antifungal capabilities against *Aspergillus niger* compared to the standard drug FLZ (fluconazole). Altogether, we identified a new series of antifungal agents.

## 1. Introduction

In recent years, the demand for the development of new antifungal drugs has increased due to increases in morbidity and associated mortality due to invasive fungal infections (IFIs) [1,2]. Recent studies have stipulated that *Candida albicans* and *Aspergillus niger* strains are responsible for the most severe pathogenic fungal infections. [3,4,5]. A major threat is the increase in the number of immunocompromised individuals who are extremely susceptible to IFIs due to either clinical treatment in intensive care units or the use of immunosuppressive medications. In addition, the erratic spread of fungal species thereby results in higher rates of drug resistance. Five categories of NMT inhibitors have been reported: (a) peptidomimetic inhibitors [6,7], (b) myristic acid analogues [8,9] (c) p-toluene sulphonamide inhibitors [10], (d) benzofurans inhibitors [11,12,13] and (e) benzothiazole inhibitors [14]. However, a stable NMT inhibitor has not yet been found. Therefore, the development of novel antifungal drugs with optimal pharmacokinetics, low levels of risk of causing drug resistance, reduced drug–drug interactions, and high levels of efficacy via novel modes of action is urgently needed [15].

**Figure 1:**
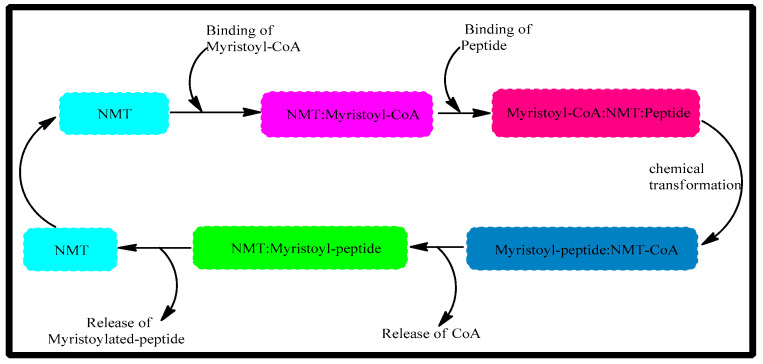
The catalytic mechanism of NMT.

Myristoyl-CoA: It has been established that the monomeric enzyme N-Myristoyltransferase (NMT) is necessary for the survival of pathogenic fungi such as *Candida albicans* [16] and *Cryptococcus neoformans* [17]. Protein N-Myristoyltransferase (NMT) catalyses myristoylation reactions via a sequential ordered Bi–Bi mechanism [18] (Figure 1). Myristoylation is described as the covalent attachment of a 14-carbon saturated fatty acid myristate to an N-terminal glycine or the lysine residues of nascent polypeptides of a variety of eukaryotic cellular and viral proteins [19]. The reaction follows the removal of N-terminal methionine residue by methionine amino-peptidase [20]. In fungi, the myristoyl group is involved in regulating the interaction between a myristoyl protein and the cellular membrane. [21,22,23]. Hence, myristoylation is a crucial process for some proteins, enabling them to localise to their intended destination and function [24]. These proteins are involved in the signal transduction cascade and vesicular and protein trafficking [25]. NMT has been found to occur in a wide variety of organisms, including mammals [26], plants [27], nematodes [28], fungi [29] and protozoans [30]. Although NMT is also present in mammalian cells, there are substantial differences in the peptide substrate specificity between human and fungal NMT, which could be utilized to avoid the unfavourable effects produced by suppressing human NMT [31,32]. Therefore, NMT is a good target for novel antifungal antibiotics.

**Figure 2:**
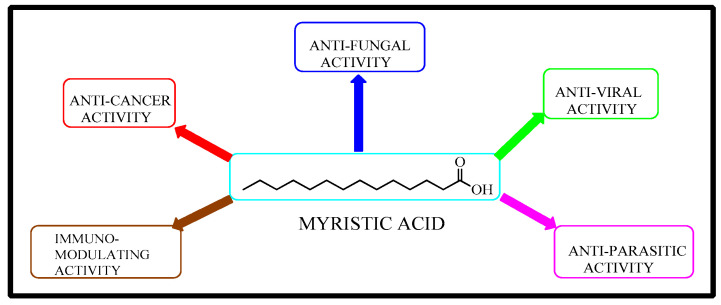
The major pharmacological activities of myristic acid.

Myristic acid is a fatty acid characterised by the presence of a carboxyl moiety (-COOH) at one end and a methyl moiety (-CH_3_) at the other end [33]. Myristic acid is a 1-tetradecanoic acid (14:0) found in human cellular membranes at low concentrations. The 14-carbon saturated fatty acid is hydrophobic and attached to lipid-anchored proteins [34]. The major pharmacological activities of myristic acid are anti-fungal, anti-viral, anti-cancer, anti-parasitic and immune-modulating activities (Figure 2) [35,36,37].

In this study, we attempted to develop novel myristic acid derivatives and investigate their potential anti-fungal properties. As part of the process, we synthesised, purified, analysed, and screened novel ligands of NMT for antifungal activity. To correlate the results, molecular docking, SAR analysis, molecular dynamic simulations and an NMT inhibition assay were carried out.

## 2. Results

### 2.1. Rational Design of Myristic Acid Derivatives

**Figure 3:**
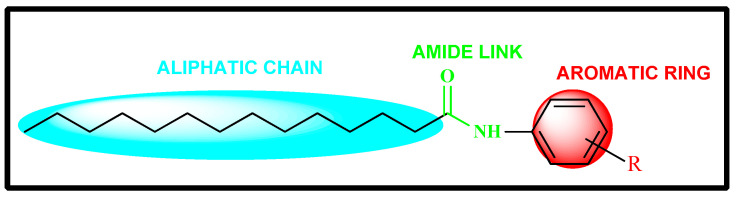
Rational design of myristic acid derivatives against NMT.

The structures of myristic acid derivatives consist of three parts (Figure 3). They each contain an aliphatic hydrocarbon chain, an amide linker and an aromatic ring attached to the amide group to enhance binding to NMT. By modifying the methods, derivatives of myristic acid were prepared that differ from those previously reported for antibacterial activity [38].

### 2.2. Synthesis

**Scheme 1:**
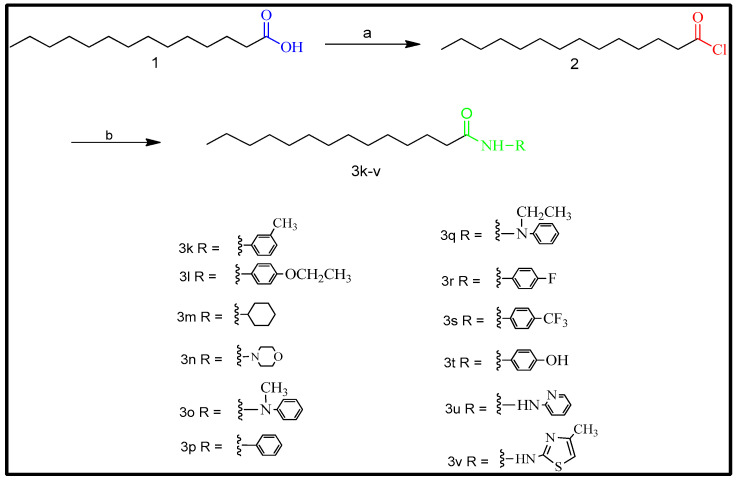
Reagents and conditions: (**a**) thionyl chloride, toluene, Δ, 62 °C, reflux, 8 h; (**b**) aryl amines, toluene, DIPEA, 25 °C, stirred, 12 h.

The synthetic route for the synthesis of myristic acid derivatives is outlined in Scheme 1. By reacting myristic acid (1) with thionyl chloride in toluene at 45 °C, the myristic acid was transformed into myristoyl chloride (2), which was subsequently subjected to an anilide synthesis procedure. The resulting products, **3k**-**3v**, were prepared by coupling compound **2** with substituted aryl amines in the presence of DIPEA as a base and toluene as a solvent at 25 °C. Using IR, ^1^HNMR, ^13^CNMR, and mass spectral techniques, the structures of the synthesized compounds were confirmed and evaluated. IR, HNMR, 13CNMR and Mass Spectra of synthesized compounds showed in Supplementary Materials.

### 2.3. High-Throughput Virtual Screening of Designed Myristic Acid Derivatives

The novel myristic acid derivatives are non-toxic to hepatocells, non-inhibitors of the CYP2D6 metabolizing enzyme and non-mutagenic and non-carcinogenic to both genders of the rat model (Table 1*).* Also, the aqueous solubility levels of all the compounds are 1 (very low) or 2 (low), with good oral bioavailability >70% (Table 2), exhibiting AlogP properties [39].

**Figure 4:**
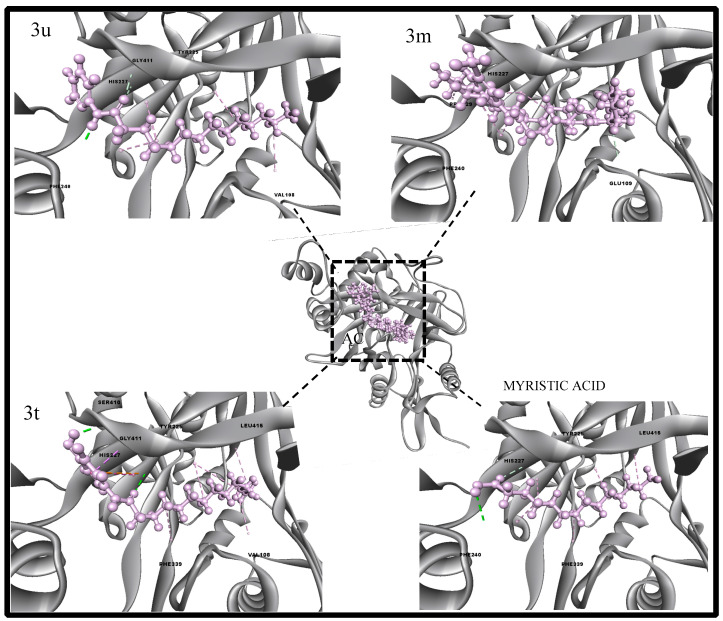
Receptor–ligand interactions of myristic acid derivatives’ interactions with N-Myristoyltransferase of *Candida albicans*.

When a compound violates the rule of five in terms of hydrophobicity, it indicates potential challenges in its absorption and distribution properties. However, it is crucial to acknowledge the unique characteristics of fatty acids and their significance in biological systems. In such cases, additional formulation strategies, such as using solubilizing agents, can be employed to improve solubility and bioavailability. It is worth noting that previous reports have demonstrated anti-tubular activities of long-chain fatty acid compounds, suggesting that limitations related to AlogP may not be restrictive in drug discovery and development. Investigations of the compounds’ binding activities via the CDOCKER interaction scoring function are favourable for all the compounds in comparison with myristic acid. In alignment with NMT experimental results, the docking interaction energy values of the top active compounds (**3u, 3m** and **3t**) are 50–48 kcal/mol, showing favourable docking scores and docking poses (Table 3). The region known as active site 2 in N-Myristoyltransferase binds with myristoyl-COA and interacts with specific amino acids, including VAL108, GLU109, ASP110, ASP112, PHE117, TYR119, ASN175, THR211, TYR225, HIS227, PHE240, TYR256, GLY409, SER410, GLY411, ASP412, GLY413, LEU415 and LEU451. This same region is utilised for the docking of myristic acid derivatives. All the compounds in this derivative series, including myristic acid, form hydrogen and hydrophobic interactions within the active site. Notably, the imidazole ring and side chain of HIS227 show favourable interactions with all the myristic acid derivatives. The compound **3u** and myristic acid interact favourably with specific active site residues, namely VAL108, TYR225, HIS227 and PHE240 and TYR225, HIS227, PHE240 and LEU415, respectively. Moreover, **3u** also interacts with the branched chains of HIS227, GLY411 and VAL108. Similarly, compound **3m** interacts with HIS227, PHE240 and GLU109, while **3t** interacts with amino acid residues such as HIS227, VAL108, GLY411, SER410 and LEU415 (Figure 4). Further, the aliphatic chain of the compound interacts to form hydrophobic interactions with aromatic amino acids (Tyr 225 and Phe 117) and branch chain amino acids (Leu 415 and Val 108); C=O, an intermediate linker, tends to form hydrogen-binding interactions, and the major functional reactive pharmacophore of the compound and attached functional moiety depict hydrogen and hydrophobic interactions. The applications for the binding interactions of the experimental studies are shown in Figure 4.

### 2.4. Molecular Dynamics Characterizations of N-Myristoyltransferase–Ligand Complexes

**Figure 5:**
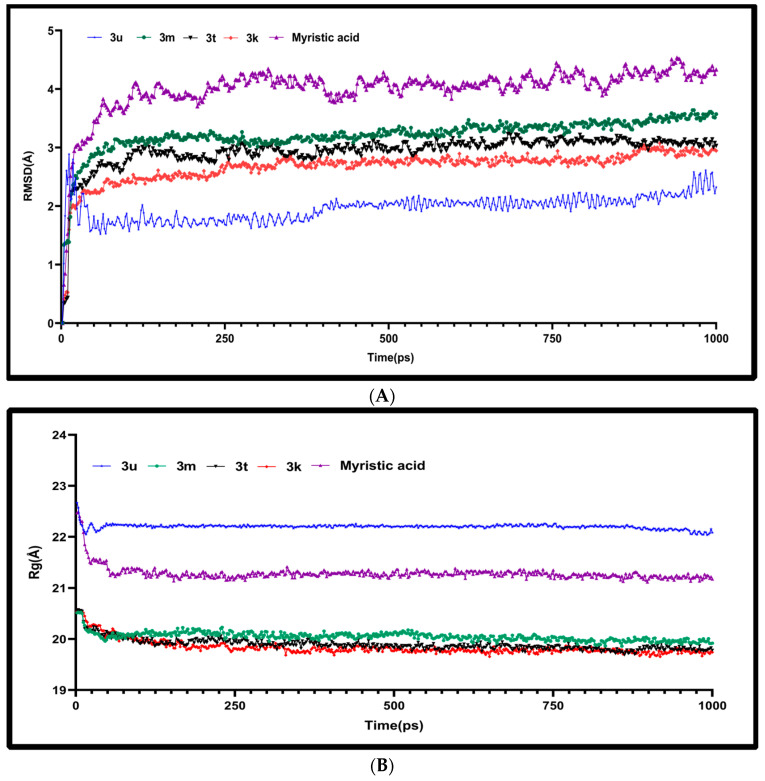
(**A**) Root mean square deviation (RMSD) of top complexes and myristic acid. **(B**) Radii of gyration (Rgs) of top complexes and myristic acid.

The dynamics and simulations of the top four complexes from the experimental analysis with ligand-bound Myristoyl-CoA in 1IYK were probed to understand the atom behaviour and the stability of each complex as a function of time. The deviations of the complexes were studied using the root mean square deviation by comparing the fluctuations from the initial to the final conformations of the protein–ligand complexes. The protein–ligand complex RMSD backbone was plotted after least-square fitting it to the backbones of all the complexes. From Figure 5A, based on the observations from the graph, it can be noted that the **3u** compound exhibits a slight increase in deviation at the beginning of its dynamics but maintains a relatively low deviation of less than 2.5 Å throughout the simulation. Similarly, the **3m, 3t** and **3k** compounds show slightly higher degrees of deviation compared to **3u,** while myristoyl-CoA exhibits a slightly higher degree of deviation than the other compounds. However, none of the conformational deviations exceed 5 Å. Furthermore, the radius of gyration (Rg) provides insight into the spatial extent and compactness of the biomolecular system. The Rg values, shown in Figure 5B, indicate that all four complexes (**3u, 3m, 3t, 3k** and myristoyl-CoA) reach a stable equilibrium during the trajectory and demonstrate relatively lower extents of structural compactness.

### 2.5. Structure Activity Relationship (SAR) Studies

**Figure 6:**
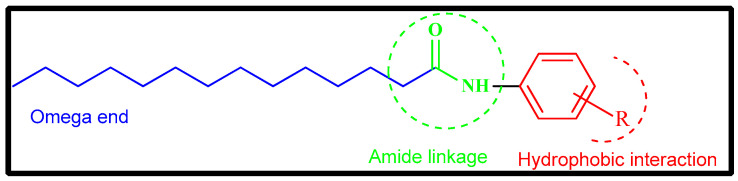
Characteristic features of synthesized compounds.

The NMT binding site interaction depends on the length and steric size of the omega-terminus of the fatty acid [40,41].The aromatic ring is believed to interact with the NMT binding site in a π–π interaction [11]. An SAR study of para-, meta- and ortho-substituted compounds showed different antifungal activities [42]. In this study, the substitutions of different functional groups on the aromatic ring influence the biological activity of compounds. In the meta and para positions, the electron-donating and hydrophobic alkyl (CH_3_) substituent (compound **3k**) shows increased antifungal activity compared to the ortho position. Interestingly, the heteroatom substituent in the aromatic ring (compound **3u**) exhibits greater antifungal activity. Hence, hydrophobic substituents are thought to increase their affinity at the receptor site. The hydroxyl (OH) functional group at the para position (compound **3t)** also shows good activity. Moreover, an aromatic ring was substituted with an electron-withdrawing group, such as -F, and exhibited moderate antifungal activity as well. Reinstating the aromatic ring with a cyclohexyl (compound **3m**) results in good activity. However, substitution with morpholinyl (compound **3n**) shows poor activity. Hence, due to the presence of an alkyl, halogen substitution on the aromatic ring increases the antifungal activity compared to non-aromatic substitution, like compound **3v**. Thus, the binding affinity, stability, and antifungal activity of the myristic acid derivatives are affected by the amide linker as well as the functional groups on the aromatic ring (Figure 6).

### 2.6. In Vitro NMT Inhibition Assay

**Figure 7:**
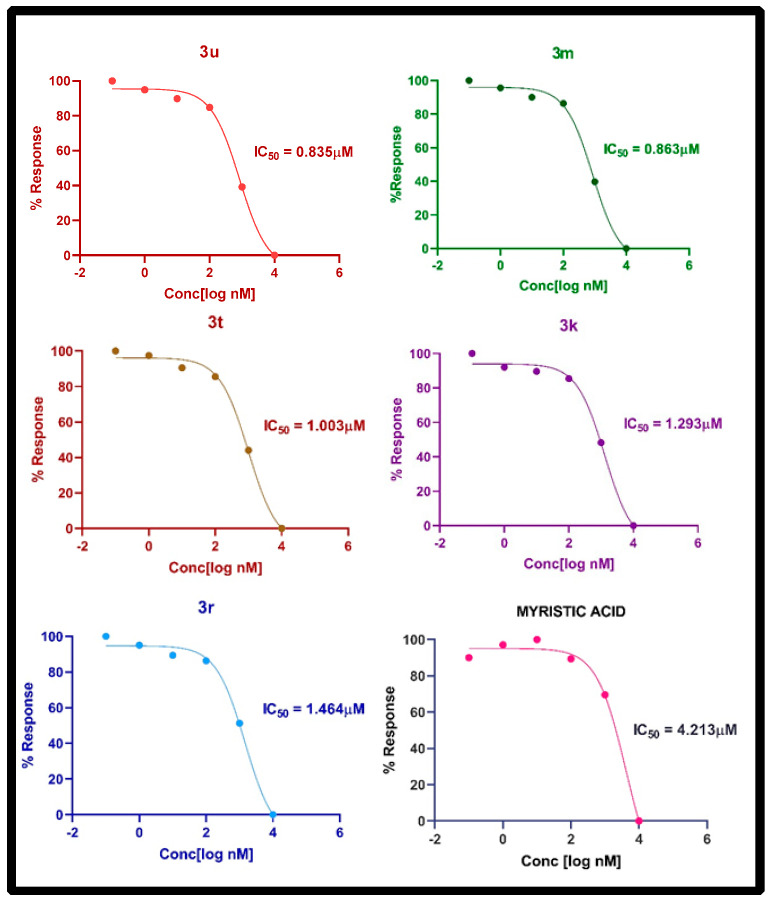
Dose-response inhibitory curves and IC_50_ values of myristic acid derivatives against myristic acid.

The potency of the myristic acid derivatives was determined via an in vitro NMT assay. Coenzyme A (CoA) was detected as a byproduct of an enzymatic reaction. Fluorescence was measured at an excitation wavelength of 380 nm and an emission wavelength of 470 nm. In this assay, the intensity of fluorescence was decreased, giving a signal (Figure 7) [43]. The compounds **3u** and **3m** demonstrated better NMT inhibition activity compared to the other synthesized compounds. The assay result indicates that the compounds **3u** and **3m** have IC_50_ values of 0.835 μM and 0.863 μM in comparison with myristic acid, which has an IC_50_ value of 4.213 μM. But **3t**, **3k** and **3r** have IC_50_ values of 1.003 μM, 1.293 μM and 1.464 μΜ, respectively. The remaining compounds have IC_50_ values > 1.500 μΜ in comparison with myristic acid. The IC_50_ values of the synthesized compounds are tabulated in Table 4.

### 2.7. Antifungal Activity

A microbroth dilution method was used to test the synthesized compounds. In vitro tests for antifungal activity against *Candida albicans* (*C. albicans*) and *Aspergillus niger* (*A. niger*) were carried out in triplicate at concentrations ranging from 100 μg/mL to 6.25 μg/mL. The percentage cytotoxicity and MIC_50_ value for each compound were calculated from the observed absorbance measured at 590 nm, and the results are shown in Table 5. Fluconazole was used as a reference drug, and the antifungal activities were expressed as minimum inhibitory concentration (MIC_50_) values in μg/mL.

**Figure 8:**
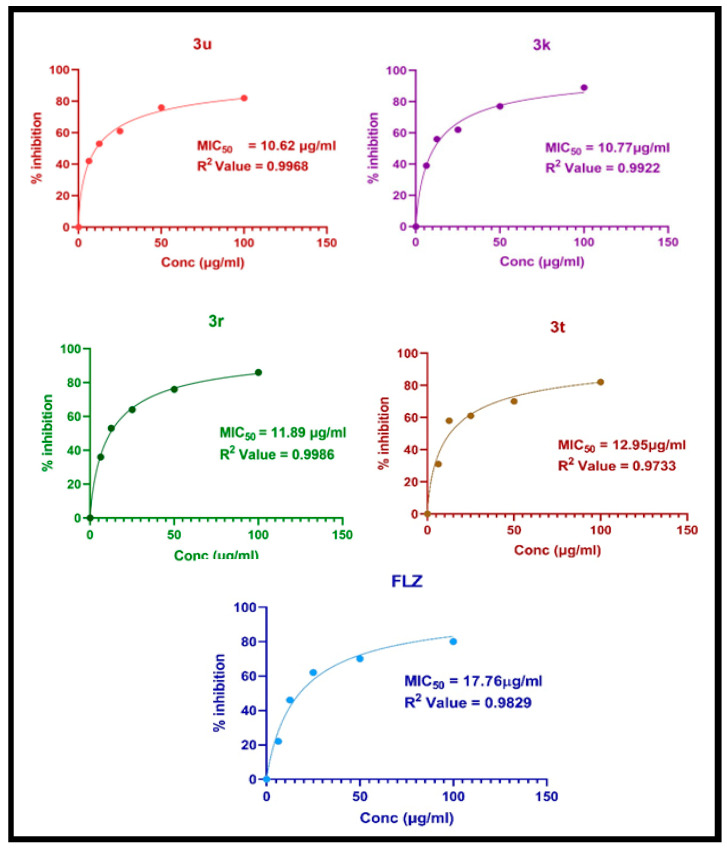
The percentage inhibition values at different concentrations of the compounds **3u**, **3k**, **3r** and **3t** and the standard drug fluconazole (**FLZ**) against *Candida albicans* and its MIC_50_.

**Figure 9:**
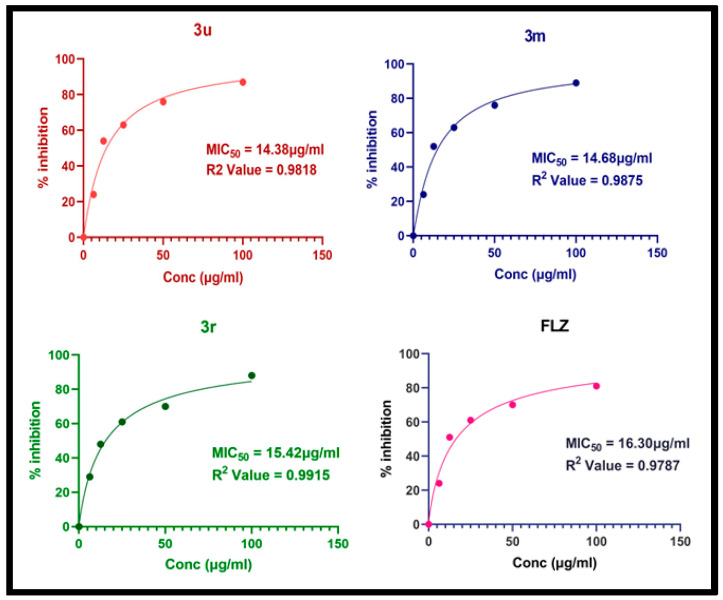
The percentage inhibition values at different concentrations of the compounds **3u**, **3m** and **3r** and the standard drug fluconazole (**FLZ**) against *Aspergillus niger* and its MIC_50_.

The in vitro antifungal activity assay in Table 5 shows that the myristic acid derivatives **3k**-**3v** have good potency against *Candida albicans* (*C. albicans*) and *Aspergillus niger* (*A. niger*) Moreover, the compounds **3u, 3k, 3r** and **3t** were more active against *Candida albicans* (MIC_50_ = 10.62, 10.77, 11.89 and 12.95 μg^.^mL^−1^) than fluconazole (MIC_50_ = 17.76 μg^.^mL^−1^). The compounds **3l, 3m, 3o** and **3p** exhibit moderate activity, with an MIC_50_ range of 15–22 μg^.^mL^−1^, against *Candida albicans* compared to fluconazole. In case of the *Aspergillus niger* (*A. niger*) strain, compound **3u** (MIC_50_ = 14.38 μg^.^mL^−1^) shows better activity than fluconazole (MIC_50_ = 16.30 μg^.^mL^−1^). Compounds **3m** and **3r** expressed good effects, with MIC_50_ values of 14.68, and 15.42 μg^.^mL^−1^ compared to fluconazole, and others show poor activities. The percentage inhibition was calculated and plotted in the graph to determine the MIC_50_ values for the compounds **3u**, **3k**, **3r** and **3t** against *Candida albicans* (Figure 8) and the compounds **3u**, **3m** and **3r** against *Aspergillus niger* (Figure 9).

## 3. Discussion

The present study aimed to develop novel antifungal agents based on their mechanisms of action. NMT is thought to be a molecular target for the forthcoming class of antifungal drugs and was therefore selected as a target [44]. Considering the structural features of myristic acid that are necessary for the myristoylation process in *Candida albicans* and *Aspergillus niger* led to the design of the myristic acid derivatives. Newly synthesized myristic acid derivatives (**3k**-**3v**) were analysed using spectroscopic methods (FTIR, ^1^HNMR, ^13^CNMR and mass spectrometry). Molecular docking studies with the target N-Myristoyltransferase (NMT), ID: 1IYK, predict that the compounds **3u**, **3m** and **3t** show good docking scores and docking poses.

All the newly synthesized myristic acid derivatives were screened in a biochemical NMT assay. In a fluorescence microplate assay, the compounds **3u**, **3m** and **3t** showed better activity compared to other compounds and myristic acid. Some authors have reported a pseudo-peptidic NMT inhibitor with an IC_50_ value of 0.50 μM [45]. Since no standard drug is available as an NMT inhibitor, we have not compared our samples with the standard drug. 

In general, antifungal activity was studied using a micro-broth dilution method. According to the antifungal activity results, the compounds **3u, 3k, 3r** and **3t** exhibit better activity against *Candida albicans*. The compounds **3u, 3m** and **3r** are also active against *Aspergillus niger* compared to the standard drug fluconazole. The presence of an alkyl, halogen substitution on the aromatic ring increased the antifungal activity compared to a non-aromatic substitution like compound **3v**. Therefore, substitution on the aromatic ring enhances antifungal activity [6]. Hence, further modification studies are required to develop a new class of antifungal agents.

## 4. Materials and Methods

### 4.1. In Silico Studies and Spectral Data Analysis

The novel derivatives of myristic acid were designed based on pharmacodynamics and pharmacokinetics requirements. BIOVIA, Discovery Studio 2019 (Dassault systems Biovia corp), was used to perform in silico predictions to comprehend the molecular behaviour. Bayesian and regression models were also used to examine the compounds’ established dosage ranges, mutagenicity, and carcinogenicity. Myristic acid (chemically pure) was purchased from Avra synthesis pvt., Ltd. (Hyderabad, India), and the other reagents were analytical pure and procured from s d fine-chem limited (Mumbai, India). The melting points of the novel myristic acid derivatives were determined in open capillary tubes using a melting point instrument and are uncorrected. Aluminium plates, precoated with silica gel G, were used to carry out TLC using n-hexane and ethyl acetate as the mobile phase (9:1). This can be visualised using UV light. Silica gel column chromatography was performed with Silica gel 60 G for the purification of the compounds. IR spectra were recorded with a Shimadzu infrared FTIR spectrophotometer (FTIR-8400S, Shimadzu, Kyoto, Japan), utilising the KBr pellet technique. ^1^H-NMR and ^13^C-NMR were measured using a Bruker-400MHz and Agilent VNMRS 400 equipment (Agilent 400 Hz, Agilent, Santa Clara, CA, USA). ^1^H-NMR chemical shifts were reported with respect to ppm ranging from 0 to 10 with an internal standard of Tetramethylsilane (TMS), and ^13^C-NMR chemical shifts were reported in parts per million relative to CDCl_3_ (77.0 ppm). Mass spectra were recorded using an ESI-MSMS (Make-Waters USS, Model-Xevo G2-XS Q TOF, Make-Waters, Illinois, IL, USA).

### 4.2. General Procedure for the Synthesis of Myristic Acid Derivatives

**Step 1**: Synthesis of myristoyl chloride from myristic acid

Myristic acid (**1**) (1 eq) was utilised as a precursor in the synthesis of myristoyl chloride, and it was melted at 45 °C in a double-necked round-bottom flask. Over the course of 30 min, drops of a thionyl chloride (4 eq) solution in toluene (30 mL) were added. The reaction mixture was refluxed at 62 °C for 8 h. The reaction was monitored via TLC using n-hexane and ethyl acetate as a mobile phase in a ratio of 9:1. A potassium permanganate solution was used as a developer. After the distillation of the solvent, myristoyl chloride (**2**) was obtained as a yellow liquid.

**Step 2**: Coupling myristoyl chloride with substituted aryl amines.

The myristoyl chloride (**2**) solution was added dropwise to a mixture of the corresponding aryl amines (0.8 eq) in toluene (25 mL) and a base of DIPEA (1.09 eq). The mixture was stirred at 25 °C for 12 h. TLC was used to monitor the reaction, with n-hexane and ethyl acetate serving as the mobile phase in a 9:1 ratio. After completion, the reaction mixture was neutralized with a 10% sodium bicarbonate (50 mL) solution and extracted using ethyl acetate (120 mL). The organic layer was washed 3 times with a 10% sodium hydroxide (30 mL) solution. Finally, it was washed with water (50 mL) and a saturated brine solution. Then, the resulting product was dried over anhydrous sodium sulfate and concentrated under reduced pressure. The residue was purified via silica gel column chromatography (CH_2_Cl_2_/MeOH= 20:1, *v*/*v*) to obtain the compounds **3k**-**3v** shown in Table 6.

#### 4.2.1. N-(m-tolyl) tetradecanamide (**3k**)

Colour: brown crystals, yield: 65.53%, melting point: 79–81 °C, molecular formula: C_21_H_35_NO. IR (KBr, cm^−1^): 3448.47 (N-H stretching), 2954.31 (Ar C-H stretching), 2919.25 (Ali C-H stretching), 1702.98 (C=O stretching), 1535.11 (Ar C=C stretching). ^1^H NMR: (CDCl_3_, 400 MHz, δ ppm): 0.885 (t, J = 7.2Hz, 3H, CH_3_), 1.281 (m, 20H, -CH_2_-), 1.650 (m, 2H, -CH_2_-), 2.340 (t, 2H, -CH_2_-), 2.378 (s, J = 7.2Hz, 3H, CH_3_), 7.175 (d, J = 8.0Hz, 2H, Ar-H), 7.261 (s, 1H, -NH), 7.395 (d, J = 8.0Hz, 2H, Ar-H). ^13^C- NMR: (CDCl_3_, 400 MHz, δ ppm): 14.085 (CH3), 21.431 (CH3), 22.680 (CH2), 24.702 (CH2), 29.072 (9 CH2), 31.920 (CH2), 34.095 (CH2), 120.634 (2ArCH), 128.755 (2ArCH), 133.851 (1ArC), 138.847 (1ArC), 179.958 (C=O). LC-MSMS (m/z) peak calculated for C_21_H_35_NO [M+1]^+^: 318.2752 peak found [M+1]^+^: 318.3335.

#### 4.2.2. N-(4-ethoxyphenyl) tetradecanamide (**3l**)

Colour: brownish crystals: yield: 61.30%, melting point: 77–80 °C, molecular formula: C_22_H_37_NO_2_. IR (KBr, cm^−1^): 3448.90 (N-H stretching), 2954.67 (Ar C-H stretching), 2919.92 (Ali C-H stretching), 1660.62 (C=O stretching), 1532.81 (Ar C=C stretching). ^1^H NMR: (CDCl_3_, 400 MHz, δ ppm): 0.873 (t, *J* = 7.2Hz, 3H, CH_3_), 1.312 (m, 22H, -CH_2_-), 1.653 (m, 2H, -CH_2_-), 2.313 (t, 2H, -CH_2_-), 2.358 (s, *J* = 7.2Hz, 3H, CH_3_), 7.283 (d, *J* = 8.0Hz, 2H, Ar-H), 7.388 (s, 1H, -NH), 7.512 (d, *J* = 8.0Hz, 2H, Ar-H). ^13^C- NMR: (CDCl_3_, 400 MHz, δ ppm): 14.070 (CH3), 22.672 (CH3), 24.724 (CH2), 25.836 (CH2), 29.346 (9 CH2), 31.915 (CH2), 37.693 (CH2), 121.075 (2ArCH), 129.551 (2ArCH), 133.851 (1ArC), 155.752 (1ArC), 179.507 (C=O). LC-MSMS (*m/z*) peak calculated for C_22_H_37_NO_2_ [M+1]^+^: 348.2858 peak found [M+1]^+^: 348.3464.

#### 4.2.3. N-cyclohexyltetradecanamide: (**3m**)

Colour: brown crystals: yield: 63.20%, melting point: 74–78 °C, molecular formula: C_20_H_39_NO. IR (KBr, cm^−1^): 3447.81 (N-H stretching), 2953.98 (Ar C-H stretching), 2918.22 (Ali C-H stretching), 1701.55 (C=O stretching), 1638.86 (Ar C=C stretching). ^1^H NMR: (CDCl_3_, 400 MHz, δ ppm): 0.878 (t, *J* = 7.2Hz, 3H, CH_3_), 1.317 (m, 24H, -CH_2_-), 1.663 (m, 2H, -CH_2_-), 2.336 (t, 2H, -CH_2_-), 2.374 (s, *J* = 7.2Hz, 3H, CH_3_), 7.109 (d, *J* = 8.0Hz, 2H, Ar-H), 7.284 (s, 1H, -NH), 7.380 (d, *J* = 8.0Hz, 2H, Ar-H). ^13^C- NMR: (CDCl_3_, 400 MHz, δ ppm): 14.250 (CH3), 20.963 (CH3), 22.821 (CH2), 25.837 (CH2), 29.534 (9 CH2), 32.054 (CH2), 37.911 (CH2), 120.075 (2ArCH), 129.551 (2ArCH), 133.851 (1ArC), 135.593 (1ArC), 171.561 (C=O). LC-MSMS (*m/z*) peak calculated for C_20_H_39_NO [M+1]^+^: 310.3065 peak found [M+1]^+^: 310.3651.

#### 4.2.4. 1-morpholinotetradeca-1-one: (**3n**)

Colour: orange crystals: yield: 65.10%, melting point: 76–78 °C, molecular Formula: C_18_H_35_NO_2_. IR (KBr, cm^−1^): 3450.04 (N-H stretching), 2954.24 (Ar C-H stretching), 2917.68 (Ali C-H stretching), 1701.50 (C=O stretching), 1471.30 (Ar C=C stretching). ^1^H NMR: (CDCl_3_, 400 MHz, δ ppm): 0.883 (t, *J* = 7.2Hz, 3H, CH_3_), 1.323 (m, 22H, -CH_2_-), 1.651 (m, 2H, -CH_2_-), 2.343 (t, 2H, -CH_2_-), 2.381 (s, *J* = 7.2Hz, 3H, CH_3_), 7.109 (d, *J* = 8.0Hz, 2H, Ar-H), 7.284 (s, 1H, -NH), 7.380 (d, *J* = 8.0Hz, 2H, Ar-H). ^13^C- NMR: (CDCl_3_, 400 MHz, δ ppm): 14.077 (CH3), 22.678 (CH3), 24.680 (CH2), 25.837 (CH2), 29.663 (9 CH2), 31.916 (CH2), 34.104 (CH2), 120.075 (2ArCH), 129.551 (2ArCH), 133.851 (1ArC), 135.593 (1ArC), 180.288 (C=O). LC-MSMS (*m/z*) peak calculated for C_18_H_35_NO_2_ [M+1]^+^: 298.2701 peak found [M+1]^+^: 298.3297.

#### 4.2.5. N-methyl-N-phenyltetradecanamide: (**3o**)

Colour: orange liquid, yield: 64.12%, melting point: 78–80 °C, molecular formula: C_21_H_35_NO. IR (KBr, cm^−1^): 3679.57 (N-H stretching), 2924.51 (Ar C-H stretching), 2854.01 (Ali C-H stretching), 1665.87 (C=O stretching), 1595.60 (Ar C=C stretching). ^1^H NMR: (CDCl_3_, 400 MHz, δ ppm): 0.882 (t, *J* = 7.2Hz, 3H, CH_3_), 1.288 (m, 20H, -CH_2_-), 1.644 (m, 2H, -CH_2_-), 2.303 (t, 2H, -CH_2_-), 2.341 (s, *J* = 7.2Hz, 3H, CH_3_), 7.159 (d, *J* = 8.0Hz, 2H, Ar-H), 7.283 (s, 1H, -NH), 7.383 (d, *J* = 8.0Hz, 2H, Ar-H). ^13^C- NMR: (CDCl_3_, 400 MHz, δ ppm): 14.059 (CH3), 22.655 (CH3), 24.801 (CH2), 25.553 (CH2), 29.565 (9 CH2), 31.894 (CH2), 34.021 (CH2), 127.245 (2ArCH), 127.709 (2ArCH), 129.679 (1ArC), 144.185 (1ArC), 178.237 (C=O). LC-MSMS (*m/z*) peak calculated for C_21_H_35_NO [M+1]^+^: 318.2752 peak found [M+1]^+^: 318.3335.

#### 4.2.6. N-phenyltetradecanamide: (**3p**)

Colour: brown crystals: yield: 60.10%, melting point: 74–76 °C, molecular formula: C_20_H_33_NO. IR (KBr, cm^−1^): 3447.69 (N-H stretching), 2954.20 (Ar C-H stretching), 2917.76 (Ali C-H stretching), 1701.27 (C=O stretching), 1601.47 (Ar C=C stretching). ^1^H NMR: (CDCl_3_, 400 MHz, δ ppm): 0.884 (t, *J* = 7.2Hz, 3H, CH_3_), 1.280 (m, 20H, -CH_2_-), 1.651 (m, 2H, -CH_2_-), 2.344 (t, 2H, -CH_2_-), 2.381 (s, *J* = 7.2Hz, 3H, CH_3_), 7.109 (d, *J* = 8.0Hz, 2H, Ar-H), 7.259 (s, 1H, -NH), 7.284 (d, *J* = 8.0Hz, 2H, Ar-H). ^13^C- NMR: (CDCl_3_, 400 MHz, δ ppm): 14.091 (CH3), 22.686 (CH3), 22.821 (CH2), 24.684 (CH2), 29.594 (9 CH2), 31.923 (CH2), 34.108 (CH2), 120.093 (2ArCH), 129.078 (2ArCH), 133.851 (1ArC), 135.593 (1ArC), 180.295 (C=O). LC-MSMS (*m/z*) peak calculated for C_20_H_33_NO [M+1]^+^: 304.2596; peak found [M+1]^+^: 304.3194.

#### 4.2.7. N-ethyl-N-phenyltetradecanamide: (**3q**)

Colour: brown crystals: yield: 61.10%, melting point: 76–79 °C, molecular formula: C_22_H_37_NO. IR (KBr, cm^−1^): 3446.84 (N-H stretching), 2954.25 (Ar C-H stretching), 2918.84 (Ali C-H stretching), 1701.97 (C=O stretching), 1594.84 (Ar C=C stretching). ^1^H NMR: (CDCl_3_, 400 MHz, δ ppm): 0.896 (t, *J* = 7.2Hz, 3H, CH_3_), 1.276 (m, 20H, -CH_2_-), 1.648 (m, 2H, -CH_2_-), 2.337 (t, 2H, -CH_2_-), 2.375 (s, *J* = 7.2Hz, 3H, CH_3_), 7.156 (d, *J* = 8.0Hz, 2H, Ar-H), 7.284 (s, 1H, -NH), 7.496 (d, *J* = 8.0Hz, 2H, Ar-H). ^13^C- NMR: (CDCl_3_, 400 MHz, δ ppm): 14.073 (CH3), 20.963 (CH3), 22.680 (CH2), 24.691 (CH2), 29.437 (9 CH2), 31.922 (CH2), 34.124 (CH2), 122.841 (2ArCH), 128.337 (2ArCH), 129.029 (1ArC), 129.612 (1ArC), 180.207 (C=O). LC-MSMS (*m/z*) peak calculated for C_22_H_37_NO [M+1]^+^: 332.2909; peak found [M+1]^+^: 332.3496.

#### 4.2.8. N-(4-fluorophenyl) tetradecanamide: (**3r**)

Colour: brown crystals: yield: 60.20%, melting point: 79–81 °C, molecular formula: C_20_H_32_FNO. IR (KBr, cm^−1^): 3444.93 (N-H stretching), 2954.32 (Ar C-H stretching), 2919.00 (Ali C-H stretching), 1703.46 (C=O stretching), 1656.36 (Ar C=C stretching). ^1^H NMR: (CDCl_3_, 400 MHz, δ ppm): 0.882 (t, *J* = 7.2Hz, 3H, CH_3_), 1.278 (m, 20H, -CH_2_-), 1.666 (m, 2H, -CH_2_-), 2.285 (t, 2H, -CH_2_-), 2.338 (s, *J* = 7.2Hz, 3H, CH_3_), 7.031 (d, *J* = 8.0Hz, 2H, Ar-H), 7.153 (s, 1H, -NH), 7.452 (d, *J* = 8.0Hz, 2H, Ar-H). ^13^C- NMR: (CDCl_3_, 400 MHz, δ ppm): 14.073 (CH3), 22.672 (CH3), 24.698 (CH2), 25.651 (CH2), 29.429 (9 CH2), 31.913 (CH2), 34.066 (CH2), 115.419 (2ArCH), 115.640 (2ArCH), 121.718 (1ArC), 121.795 (1ArC), 179.846 (C=O). LC-MSMS (*m/z*) peak calculated for C_20_H_32_FNO [M+1]^+^: 322.2501; peak found [M+1]^+^: 322.3115.

#### 4.2.9. N-(4-(trifluoromethyl)phenyl)tetradecanamide: (**3s**)

Colour: brownish crystals, yield: 61.10%, melting point: 77–79 °C, molecular formula: C_21_H_32_F_3_NO. IR (KBr, cm^−1^): 3445.46 (N-H stretching), 2954.92 (Ar C-H stretching), 2918.82 (Ali C-H stretching), 1672.28 (C=O stretching), 1530.34 (Ar C=C stretching). ^1^H NMR: (CDCl_3_, 400 MHz, δ ppm): 0.884 (t, *J* = 6.8Hz, 3H, CH_3_), 1.278 (m, 20H, -CH_2_-), 1.655 (m, 2H, -CH_2_-), 2.346 (t, 2H, -CH_2_-), 2.414 (s, *J* = 7.2Hz, 3H, CH_3_), 7.284 (d, *J* = 8.0Hz, 2H, Ar-H), 7.573 (s, 1H, -NH), 7.674 (d, *J* = 8.8Hz, 2H, Ar-H). ^13^C- NMR: (CDCl_3_, 400 MHz, δ ppm): 14.092 (CH3), 22.681 (CH3), 25.575 (CH2), 29.349 (9 CH2), 31.916 (CH2), 37.994 (CH2), 119.763 (2ArCH), 125.424 (2ArCH), 126.266 (1ArC), 141.005 (1ArC), 172.027 (C=O). LC-MSMS (*m/z*) peak calculated for C_21_H_32_F_3_NO [M^_^1]: 371.2436; peak found [M^_^1]: 370.3534.

#### 4.2.10. N-(4-hydroxyphenyl) tetradecanamide: (**3t**)

Colour: brown crystals, yield: 60.15%, melting point: 77–79 °C, molecular formula: C_20_H_33_NO_2_. IR (KBr, cm^−1^): 3448.74 (N-H stretching), 2955.53 (Ar C-H stretching), 2918.53 (Ali C-H stretching), 1654.67 (C=O stretching), 1529.53 (Ar C=C stretching). ^1^H NMR: (CDCl_3_, 400 MHz, δ ppm): 0.882 (t, *J* = 7.2Hz, 3H, CH_3_), 1.277 (m, 20H, -CH_2_-), 1.667 (m, 2H, -CH_2_-), 2.342 (t, 2H, -CH_2_-), 2.379 (s, *J* = 7.2Hz, 3H, CH_3_), 7.001 (d, *J* = 8.0Hz, 2H, Ar-H), 7.031 (s, 1H, -NH), 7.606 (d, *J* = 8.0Hz, 2H, Ar-H). ^13^C- NMR: (CDCl_3_, 400 MHz, δ ppm): 14.103 (CH3), 20.963 (CH3), 22.686 (CH2), 24.711 (CH2), 29.437 (9 CH2), 31.921 (CH2), 34.024 (CH2), 120.075 (2ArCH), 129.551 (2ArCH), 133.851 (1ArC), 135.593 (1ArC), 179.501 (C=O). LC-MSMS (*m/z*) peak calculated for C_20_H_33_NO_2_ [M+1]^+^: 320.2545; peak found [M+1]^+^: 320.3158.

#### 4.2.11. N-(pyridine-2-yl) tetradecanamide: (**3u**)

Colour: yellowish crystals, yield: 62.20%, melting point: 76–78 °C, molecular formula: C_19_H_32_N_2_O. IR (KBr, cm^−1^): 3436.43 (N-H stretching), 2955.00 (Ar C-H stretching), 2918.11 (Ali C-H stretching), 1697.79 (C=O stretching), 1577.78 (Ar C=C stretching). ^1^H NMR: (CDCl_3_, 400 MHz, δ ppm): 0.872 (t, *J* = 7.2Hz, 3H, CH_3_), 1.268 (m, 20H, -CH_2_-), 1.639 (m, 2H, -CH_2_-), 2.331 (t, 2H, -CH_2_-), 2.369 (s, *J* = 7.2Hz, 3H, CH_3_), 7.284 (d, *J* = 8.0Hz, 2H, Ar-H), 7.308 (s, 1H, -NH), 7.358 (d, *J* = 8.0Hz, 2H, Ar-H). ^13^C- NMR: (CDCl_3_, 400 MHz, δ ppm): 14.082 (CH3), 20.963 (CH3), 22.675 (CH2), 24.699 (CH2), 29.392 (9 CH2), 31.913 (CH2), 34.114 (CH2), 120.075 (2ArCH), 129.551 (2ArCH), 133.851 (1ArC), 135.593 (1ArC), 180.017 (C=O). LC-MSMS (*m/z*) peak calculated for C_19_H_32_N_2_O [M+1]^+^: 305.2548; peak found [M+1]^+^: 305.3128.

#### 4.2.12. N-(4-methylthiazol-2-yl) tetradecanamide: (**3v**)

Colour: yellowish crystals, yield: 63.20%, melting point: 74–76 °C, molecular formula: C_18_H_32_N_2_OS. IR (KBr, cm^−1^): 3315.74 (N-H stretching), 2953.12 (Ar C-H stretching), 2918.40 (Ali C-H stretching), 1656.91 (C=O stretching), 1523.82 (Ar C=C stretching). ^1^H NMR: (CDCl_3_, 400 MHz, δ ppm): 0.856 (t, *J* = 6.8Hz, 3H, CH_3_), 1.352 (m, 20H, -CH_2_-), 1.665 (m, 2H, -CH_2_-), 2.263 (t, 2H, -CH_2_-), 2.446 (s, *J* = 7.2Hz, 3H, CH_3_), 7.028 (d, *J* = 8.0Hz, 2H, Ar-H), 7.272 (s, 1H, -NH). ^13^C- NMR: (CDCl_3_, 400 MHz, δ ppm): 14.091 (CH3), 22.662 (CH3), 25.036 (CH2), 29.346 (9 CH2), 31.895 (CH2), 36.128 (CH2), 127.047 (2ArCH), 132.554 (1ArC), 158.745 (1ArC), 171.276 (C=O). LC-MSMS (*m/z*) peak calculated for C_18_H_32_N_2_OS [M+1]^+^: 325.2269; peak found [M+1]^+^: 325.2711.

### 4.3. Computational Studies

In this work, the concept of a structure-based drug was implemented to probe the binding of the N-Myristoyltransferase of *Candida albicans* with the newly synthesised myristic acid derivatives using a molecular docking approach. The X-ray crystallography structure of the drug target N-Myristoyltransferase (Sogabe) from PDB (Protein databank) ID: 1IYK was retrieved from the RCSB Protein Data Bank (RCSB PDB) repository (https://www.rcsb.org/, accessed on 1 June 2023). The structures of chemical compounds were drawn and geometrically cleaned using Chem Draw Ultra (version), and the induvial structures were saved in mol format. Both the drug target and the ligands were prepared using the protein and ligand preparation wizard in BIOVIA Discovery studio 2020. The protocol of CDOCKER (Wu), a grid-based molecular dynamics docking function, was validated using a bound nonpeptidic inhibitor (MIM) to obtain the pose RMSD deviations within 2.5–3 Å. The optimized method was used to test the myristic acid derivatives and myristic acid at the same active site binding of MIM. The grid was placed at the active site along with water molecules at the 3D Cartesian space coordinates of 25.7586X, 49.4463Y and 34.8952Z. Consequently, the interpretation was based on CDOCKER’s scoring function for the interaction energy of the binding interaction. Further, ADMET, TOPKAT and Lipinski’s rule of five (RO5) were used to understand the bioavailability and toxicity profiles of the compounds using the small molecule tools in Discovery studio [46].

### 4.4. Molecular Dynamics Simulations

To further evaluate the stability and real-time behaviour of the N-Myristoyltransferase docked complex dynamics, a CHARMm simulation for 1000ps in was employed. The CHARMm heterogenous force field parameter was implemented in the BIOVIA Discovery studio suite and was applied to five complexes, such as 3u- N-Myristoyltransferase, 3m- N-Myristoyltransferase, 3t- N-Myristoyltransferase, 3k- N-Myristoyltransferase and myristic acid- N-Myristoyltransferase. The complex system of protein–ligand interactions was solvated with a TIP3 water model in an orthorhombic box and neutralized with 0.145 M of NaCl (sodium chloride). The three different complexes were processed for two stages of 500 steps of energy minimization with the steepest decent and conjugate gradient. The system was gradually heated and equilibrated and production occurred under an NPT isothermal–isobaric ensemble. A Nosé–Hoover thermostat was used as a temperature control to set the temperature at 300 k, and the pressure was fixed at 1.0 bar with a Langevin piston. The nonbond lower cutoff distance to higher cutoff distance range of 12–10 Å included with the Leapfrog Verlet Integrator was used as a dynamics integrator to perform a numerical integration of the equation of motion. The time-dependent parameter RMSD and radius of gyration were calculated to understand the stability of the complexes [47].

### 4.5. Screening Myristic Acid Derivatives Using Biochemical NMT Assay

The NMT protein used in the study was from the supplier Ray Biotech. The assay was performed in 96-well black PP microplates with the myristic acid derivatives in concentrations ranging from 0.1 nM to 10,000 nM. The solutions were prepared in a buffer containing 20 mM of potassium phosphate (pH 7.9–8.0) and 0.5 mM of ethylenediaminetetraacetic acid (EDTA), 0.1% (*v*/*v*) Triton X-100, and a final concentration of 2.7% (*v*/*v*) dimethyl sulfoxide (DMSO). Fluorescence was measured at an excitation wavelength of 380 nm and an emission wavelength of 470 nm. These readings were obtained on an EnSight Multimode Microplate Reader, and we plotted the response vs. concentration (log nM), ranging from 0.1 nM to 10,000 nM, to obtain the IC_50_ values using Graph Pad Prism, according to the reported standard procedure [43].

### 4.6. Screening for Antifungal Activity

Strains

From HIMEDIA M210 Mumbai, India, test fungal strains were acquired. The conventional micro-broth dilution method was used to test the in vitro antifungal activity of the synthesized compounds against the fungi *Candida albicans* (ATCC 10231) and *Aspergillus niger* (ATCC 16888) from the American Type Culture Collection. The acquired fungal strains were used in the current experiment after being subcultured in SDB (Sabouraud dextrose broth). The 48 h young cultures were created and kept at 26 °C before the experiment.

Antifungal Assay

According to the CLSI’s standard M27 recommended methodology, the SDB was treated with the compounds, which were dissolved in DMSO via the serial dilution method in sterile circumstances to achieve the necessary concentration levels. All the cultures that had been exposed to the compounds were serially diluted twice in growth media, inoculated to about 1 × 10^5^ CFU/mL and incubated at 26 °C for 48 h. Subsequently, using a UV spectrophotometer, the absorbance at 590 nm of each culture was determined. The formula was used to compute the viability percentage [48].

Percentage viability = (OD control−OD sample/OD control) × 100

The percentage inhibition was calculated using the formula (100 - cell viability) and plotted in the graph to determine the MIC_50_ value for each compound (Figure 8) and (Figure 9).

Statistical Analysis

GraphPad Prism 8.0.2 software (San Diego, CA, USA) was used for statistical analysis. The MIC_50_ value was established via a nonlinear regression analysis.

## 5. Conclusions

A new series of myristic acid derivatives were synthesized, analysed, screened in an NMT inhibition assay, and evaluated for their antifungal activity. Among the novel derivatives of myristic acid, the compounds **3u** and **3m** show superior NMT inhibition activity compared to myristic acid. Flexible molecular docking was used to investigate the binding mechanisms of the compounds. The results show how crucial hydrogen bonds between the aromatic ring and NMT are required for the inhibitor’s correct orientation. Thus, the results of the in silico studies predicted favourable ADMET and drug-like parameters, which can be used as a solid starting point for future research on the design and synthesis of NMT inhibitors.

## Figures and Tables

**Table 1:** The newly synthesised myristic acid derivatives and their ADMET and Topkat predictions, achieved via computational tools ^a^.

Compound	Solubility	Hepatotoxicity	CYP2D6	NTP_Rat		Ames Mutagen
				Male	Female	
**3k**	2	NT	NI	NC	NC	NM
**3l**	2	NT	NI	NC	NC	NM
**3m**	2	NT	I	NC	NC	NM
**3n**	2	NT	NI	NC	NC	NM
**3o**	2	NT	NI	NC	NC	NM
**3p**	2	NT	NI	NC	NC	NM
**3q**	1	NT	NI	NC	NC	NM
**3r**	2	NT	NI	NC	NC	NM
**3s**	1	NT	NI	NC	NC	NM
**3t**	2	NT	NI	NC	NC	NM
**3u**	2	NT	NI	NC	NC	NM
**3v**	2	NT	NI	NC	NC	NM
**Myristic acid**	2	NT	NI	NC	NC	NM

^a^ NT: non-toxic, I: inhibitor NI: non-inhibitor, NC: non-carcinogen, NM: non-mutagen.

**Table 2:** RO5 violations and set dosage toxicity predictions for myristic acid derivatives, determined using TOPKAT in Discovery studio ^a^.

Compound	Alog p	MW	HBA	HBD	Rat Oral Dose	Rat Carcinogenic Potency
					LD50 (g/kg_body_weight)	TD50 (mg/kg_body_weight/day)
**3k**	7.12	317.50	2	1	14.98	886.91
**3l**	6.96	347.53	3	1	8.07	887.65
**3m**	6.91	309.53	2	1	11.63	04.67
**3n**	4.95	297.47	3	0	9.63	49.60
**3o**	6.84	317.50	2	0	2.58	205.15
**3p**	6.63	303.48	2	1	8.96	2761.99
**3q**	7.19	331.53	2	0	7.68	777.07
**3r**	6.84	321.47	2	1	4.90	304.41
**3s**	7.57	371.48	2	1	6.15	289.64
**3t**	6.39	319.48	3	2	8.03	2336.46
**3u**	6.02	304.47	3	1	4.31	753.33
**3v**	5.91	324.52	3	1	6.04	400.96
**Myristic acid**	5.48	228.371	2	1	5.97	233.87

^a^ MW: molecular weight, HBA: hydrogen bond acceptors, HBD: hydrogen bond donors.

**Table 3:** Molecular docking scoring functions of myristic acid derivatives.

Compound	CDOCKER Interaction Energy
**3k**	48.98
**3l**	44.81
**3m**	45.14
**3n**	48.09
**3o**	45.52
**3p**	47.63
**3q**	42.76
**3r**	48.88
**3s**	41.78
**3t**	44.16
**3u**	50.34
**3v**	41.34
**Myristic acid**	36.76

**Table 4:** IC_50_ values of myristic acid derivatives using biochemical NMT assay.

Sl. No	Compound Name	IC_50_ (μM)
1	**3k**	1.293
2	**3l**	>1.500
3	**3m**	0.863
4	**3n**	>1.500
5	**3o**	>1.500
6	**3p**	>1.500
7	**3q**	>1.500
8	**3r**	1.464
9	**3s**	>1.500
10	**3t**	1.003
11	**3u**	0.835
12	**3v**	>1.500
Myristic acid	-	4.213

**Table 5:** Antifungal activities of the titled compounds against *C. albicans* and *A. niger*. (MIC_50_, μg^.^mL^−1^).

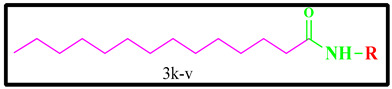			
**Compounds**	**R**	***C. albicans***	***A. niger***
**3k**	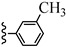	10.77	59.57
**3l**	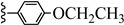	21.74	75.89
**3m**	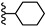	17.31	14.68
**3n**	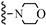	27.61	31.44
**3o**	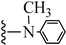	17.37	94.15
**3p**	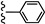	15.20	24.75
**3q**	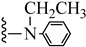	28.40	28.54
**3r**	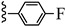	11.89	15.42
**3s**	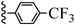	38.78	34.30
**3t**	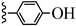	12.95	17.41
**3u**	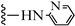	10.62	14.38
**3v**	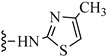	32.38	22.15
FLZ	-	17.76	16.30

*C. albicans*, *Candida albicans* (ATCC 10231); *A. niger*, *Aspergillus niger* (ATCC 16888); FLZ, Fluconazole.

**Table 6:** Physicochemical parameters of synthesized compounds.

Compound	Chemical Structure	M.Formula	Mol.wt	RF Value
**3k**	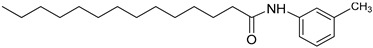	C_21_H_35_NO	318.27	0.59
**3l**	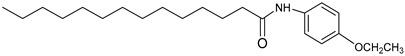	C_22_H_37_NO_2_	348.53	0.53
**3m**	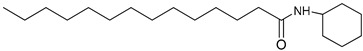	C_20_H_39_NO	310.36	0.44
**3n**	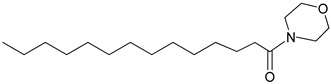	C_18_H_35_NO_2_	298.32	0.40
**3o**	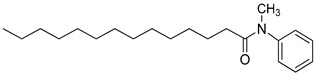	C_21_H_35_NO	318.51	0.42
**3p**	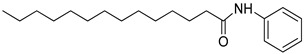	C_20_H_33_NO	304.31	0.41
**3q**	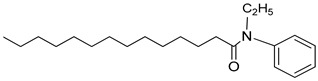	C_22_H_37_NO	332.34	0.51
**3r**	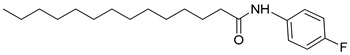	C_20_H_32_FNO	322.31	0.54
**3s**	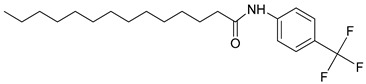	C_21_H_32_F_3_NO	370.35	0.51
**3t**	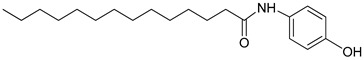	C_20_H_33_NO_2_	320.31	0.56
**3u**	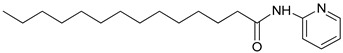	C_19_H_32_N_2_O	305.31	0.59
**3v**	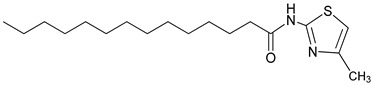	C_18_H_32_N_2_OS	325.27	0.61

TLC mobile phase: n-hexane and ethyl acetate in a ratio of 9:1. The spectral data of the newly synthesized myristic acid derivatives.

## Data Availability

Not applicable.

## Supplementary Materials

The supporting information can be downloaded at: https://www.mdpi.com/article/10.3390/antibiotics12071167/s1, Figures S1–S12: IR, ^H^NMR, ^13^CNMR and mass spectra of compounds 3k–3v.

## Sample Availability

Samples of the compounds are available from the authors.
