# Supplementary material for: Discovery of Novel Myristic Acid Derivatives as N-Myristoyltransferase Inhibitors: Design, Synthesis, Analysis, Computational Studies and Antifungal Activity
Source: Antibiotics (Basel). 2023 Jul 9;12(7):1167. doi: 10.3390/antibiotics12071167 (PMC10376843; doi:10.3390/antibiotics12071167)
Supplement: Supplementary file 1 [file antibiotics-12-01167-s001.zip › antibiotics-2475736-SI.pdf]

Discovery of novel myristic acid derivatives as *N*-Myristoyltransferase inhibitors: Design, Synthesis, analysis, computational studies and antifungal activity

Saleem Javid [1,2], Hissana Ather [3], Umme Hani [4], Ayesha Siddiqua [5], Shaik Mohammaad Asif Ansari [5], Dhivya Shanmugarajan [2], Honnavalli Yogish Kumar [2], Rajaguru Arivuselvam [6], Madhusudan N Purohit [2] and Prashantha Kumar BR [2,*]

1. Department of Pharmaceutical Chemistry, Farooqia College of Pharmacy, Mysore, 570 015, Karnataka, India
2. Department of Pharmaceutical Chemistry, JSS College of Pharmacy, Mysore, JSS Academy of Higher Education & Research, Mysore 570 015, Karnataka, India
3. Department of Pharmaceutical Chemistry, College of Pharmacy, King Khalid University, Abha 62529, Saudi Arabia
4. Department of Pharmaceutics, College of Pharmacy, King Khalid University, Abha 62529, Saudi Arabia
5. Department of Clinical Pharmacy, College of Pharmacy, King Khalid University, Abha 62529, Saudi Arabia
6. Department of Pharmaceutical Biotechnology, JSS College of Pharmacy, Mysore, JSS Academy of Higher Education & Research, Mysore 570 015, Karnataka, India.

* Correspondence: E-mail: brprashanthkumar@jssuni.edu.in; Tel. No.: +91-741-101-1124.

Supplementary Materials

Contents Page. No

1. IR, ^{H}NMR, ^{13}CNMR and Mass Spectra of synthesized compounds………………. 2 - 25

Spectra of synthesized compounds

Figure S1: IR, ^{H}NMR, ^{13}CNMR and Mass Spectra of compound 3k

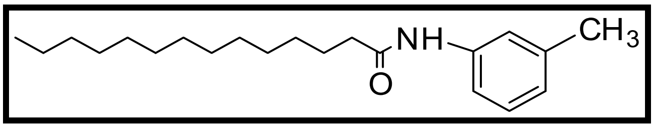

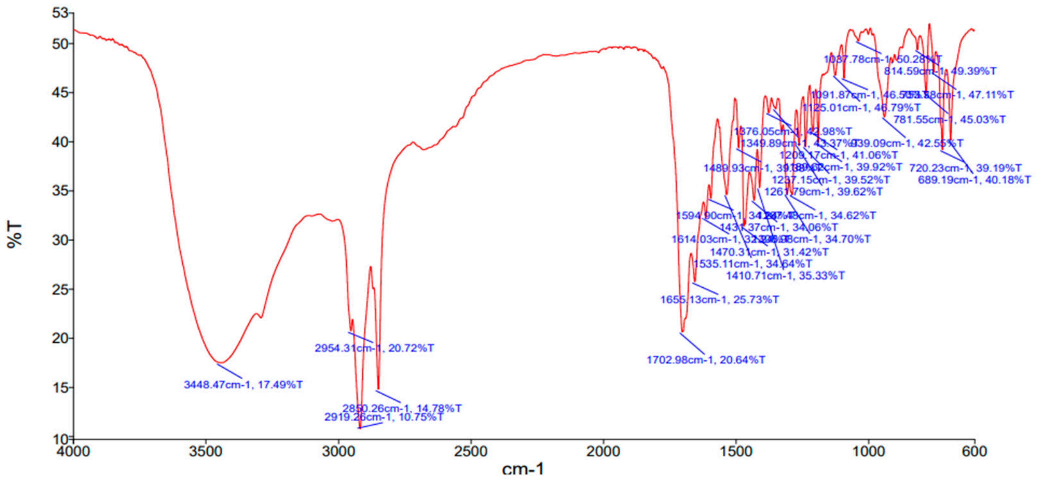

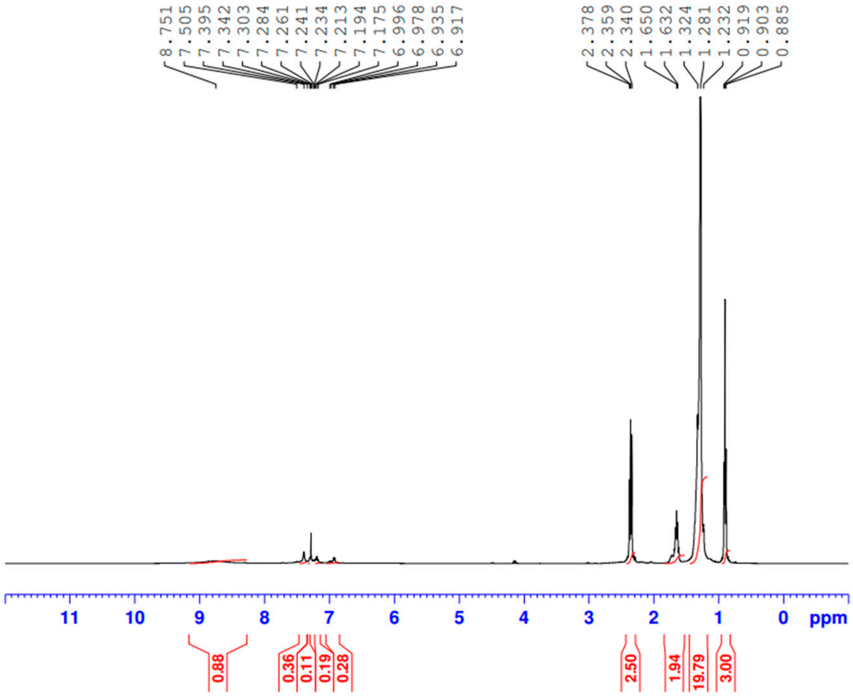

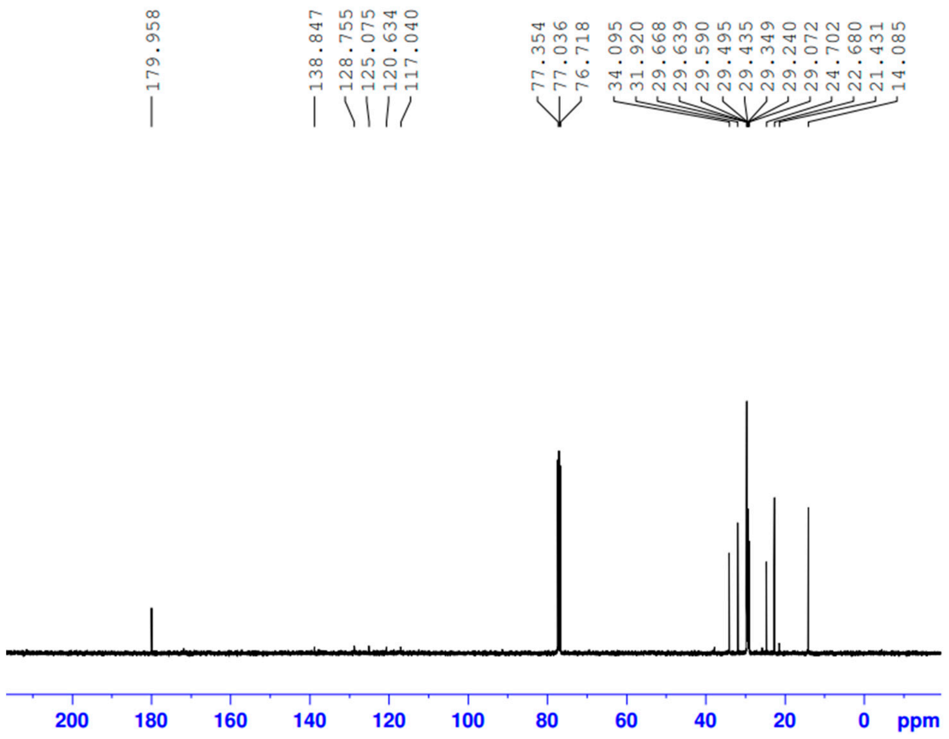

179.958
138.847
128.755
125.075
120.634
117.040
77.354
77.036
76.718
34.095
31.920
29.668
29.639
29.590
29.495
29.435
29.349
29.240
29.072
24.702
22.680
21.431
14.085
200
180
160
140
120
100
80
60
40
20
0
ppm

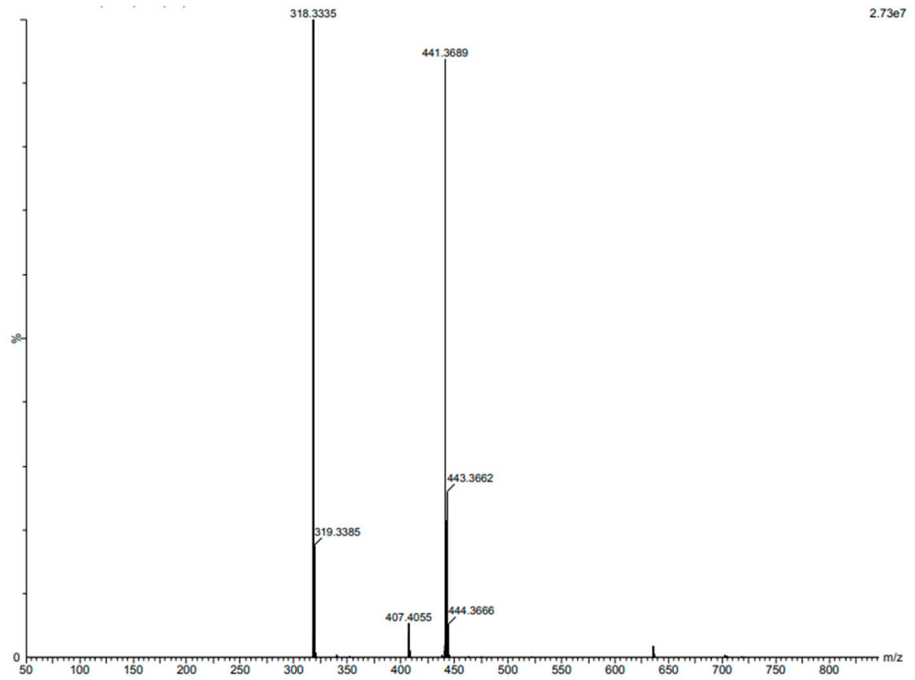

318.3335
2.73e7
441.3689
%
443.3662
319.3385
407.4055
444.3666
0
50
100
150
200
250
300
350
400
450
500
550
600
650
700
750
800
m/z

Figure S2: IR, ^{H}NMR, ^{13}CNMR and Mass Spectra of compound 31

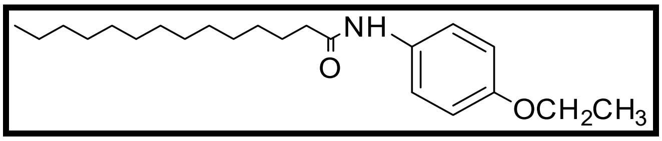

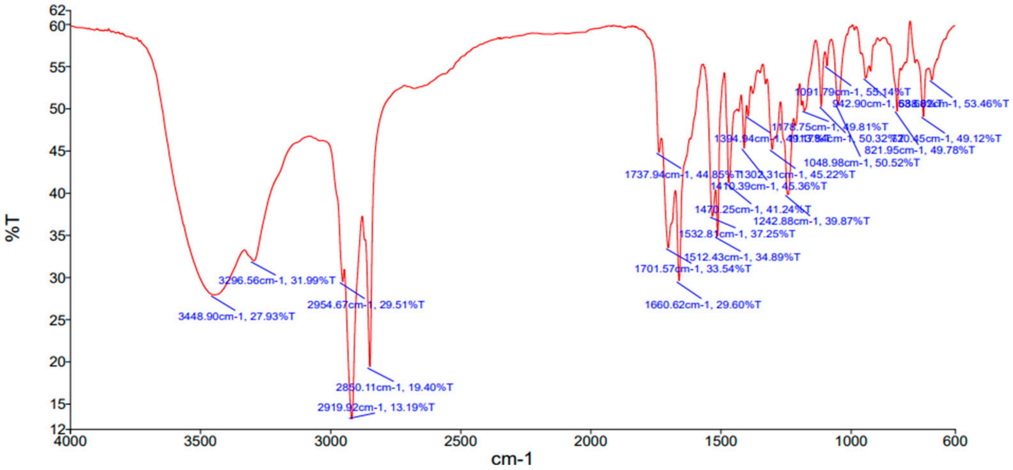

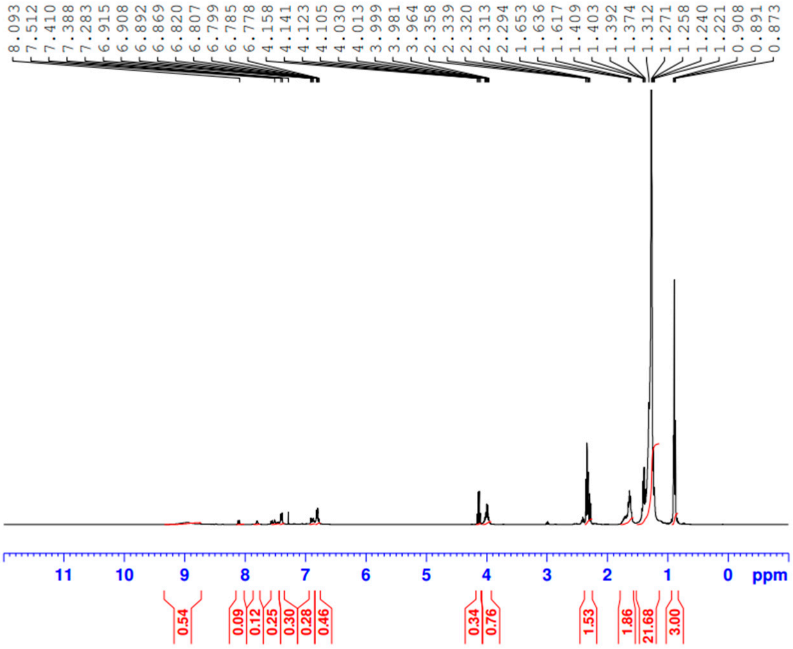

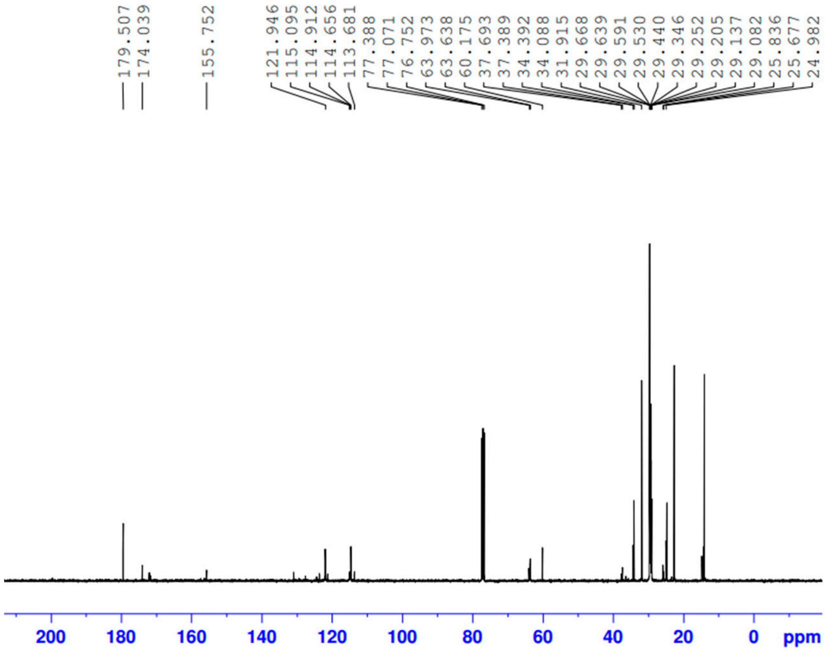
179.507
174.039
155.752
121.946
115.095
114.912
114.656
113.681
77.388
77.071
76.752
63.973
63.638
60.175
37.693
37.389
34.392
34.088
31.915
29.668
29.639
29.591
29.530
29.440
29.346
29.252
29.205
29.137
29.082
25.836
25.677
24.982
200
180
160
140
120
100
80
60
40
20
0
ppm

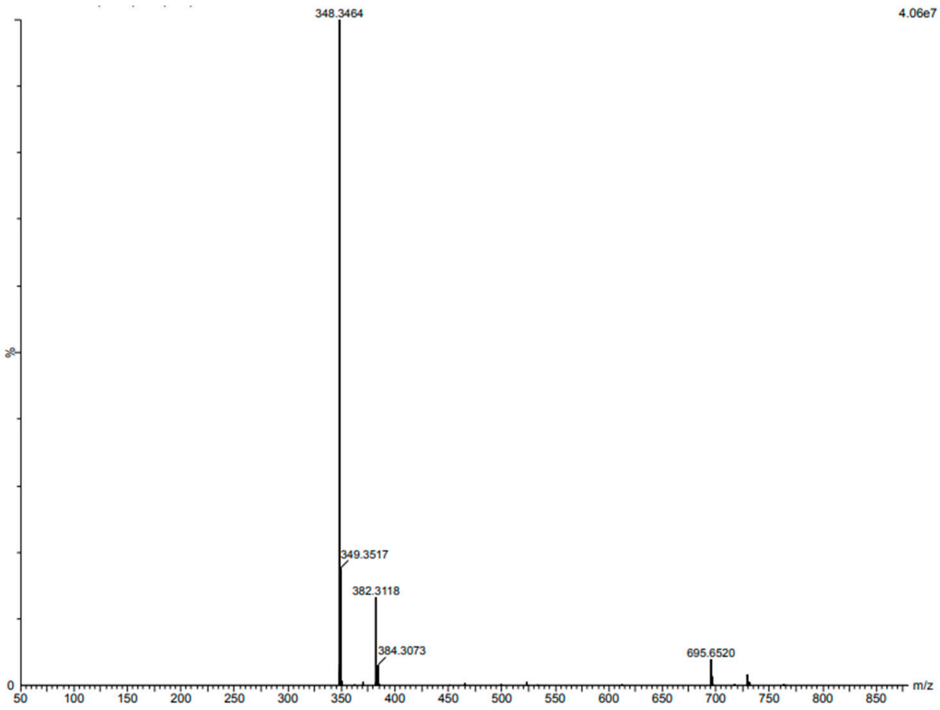
348.3464
4.06e7
%
349.3517
382.3118
384.3073
695.6520
0
50
100
150
200
250
300
350
400
450
500
550
600
650
700
750
800
850
m/z

Figure S3: IR, HNMR, 13CNMR and Mass Spectra of compound 3m

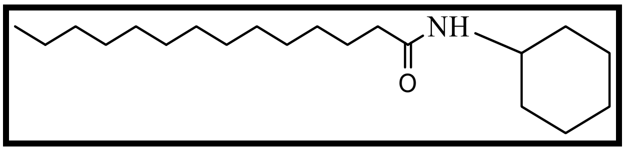

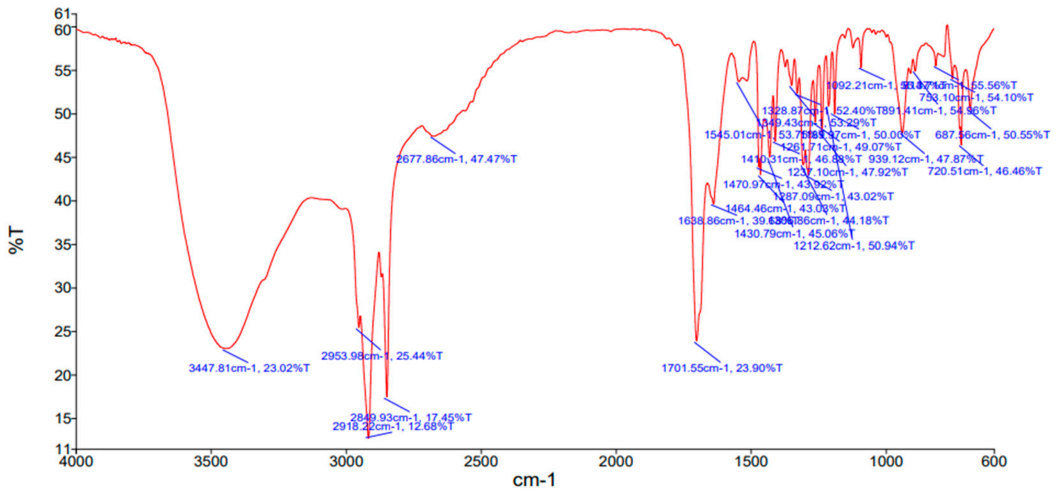

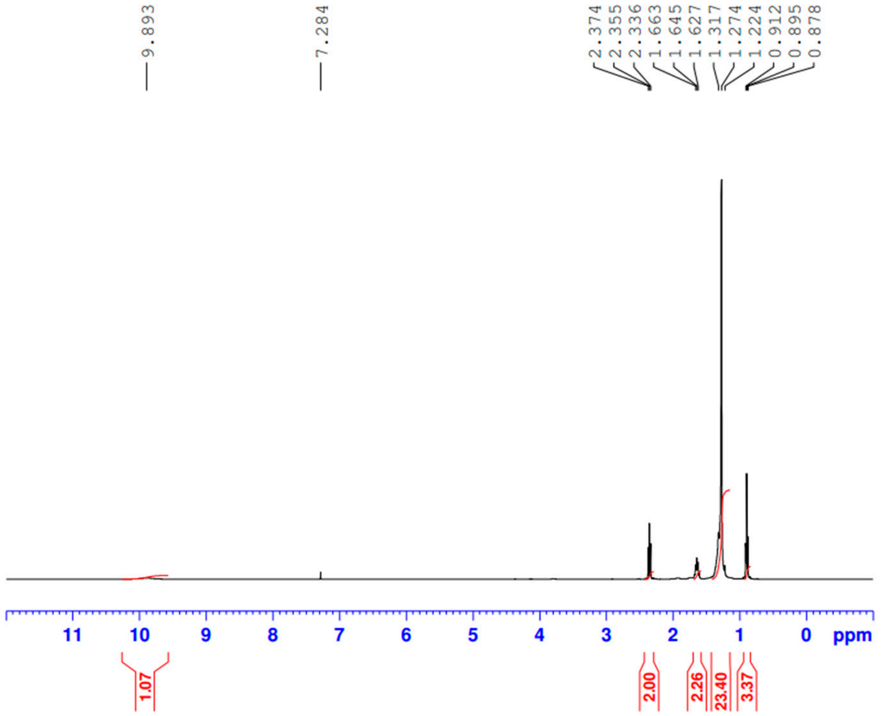

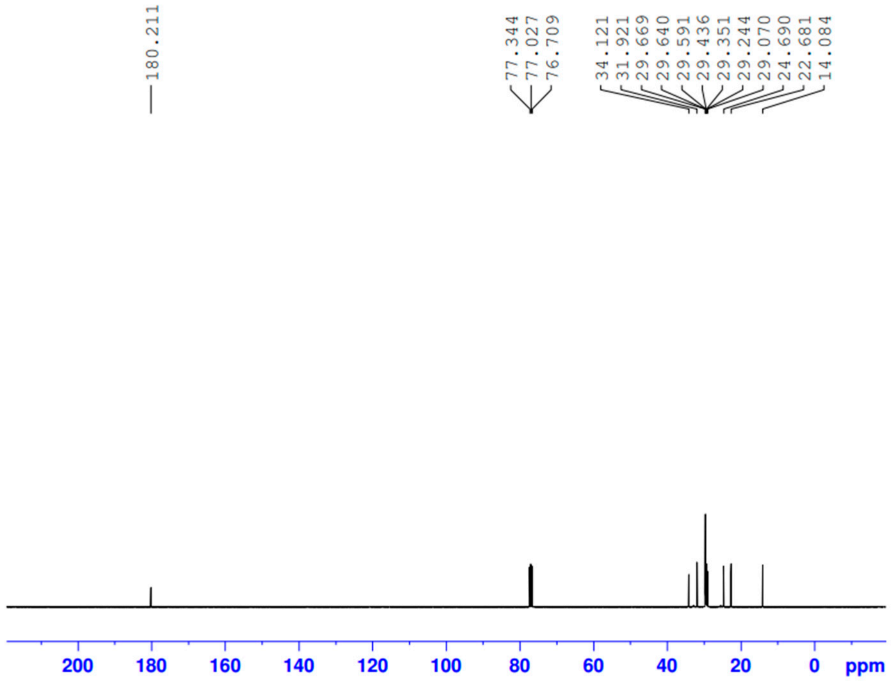
180.211
77.344
77.027
76.709
34.121
31.921
29.669
29.640
29.591
29.436
29.351
29.244
29.070
24.690
22.681
14.084
200
180
160
140
120
100
80
60
40
20
0
ppm

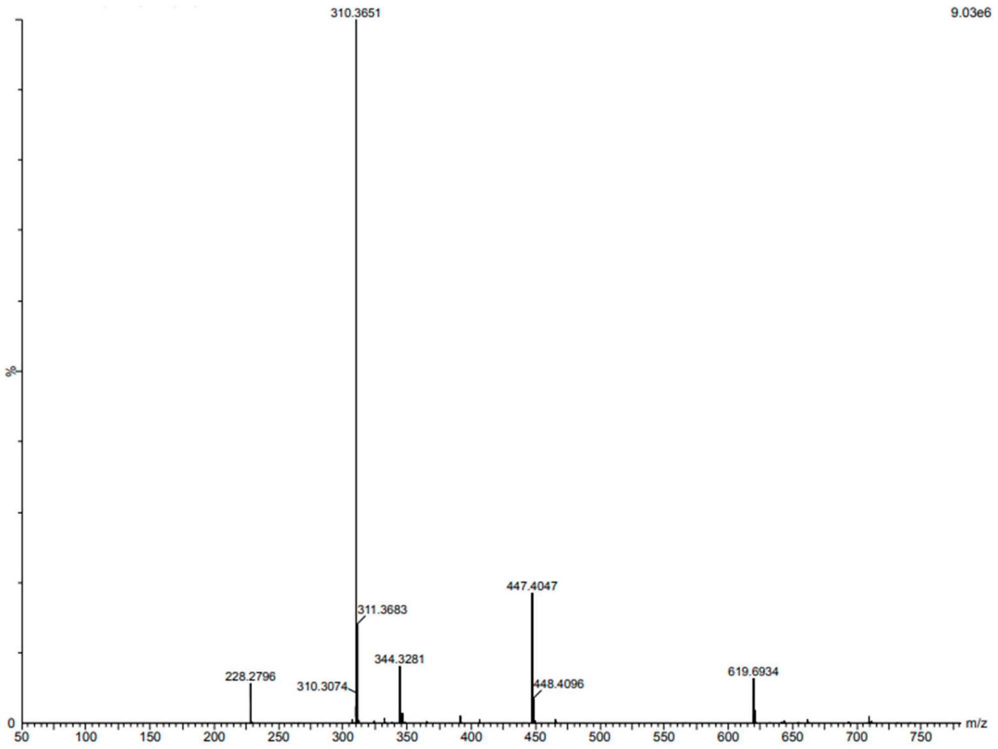
310.3651
9.03e6
%
311.3683
447.4047
344.3281
228.2796
310.3074
448.4096
619.6934
m/z

Figure S4: IR, ^{H}NMR, ^{13}CNMR and Mass Spectra of compound 3n

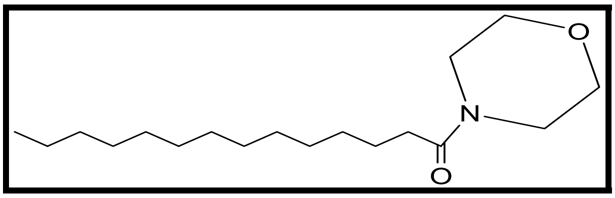

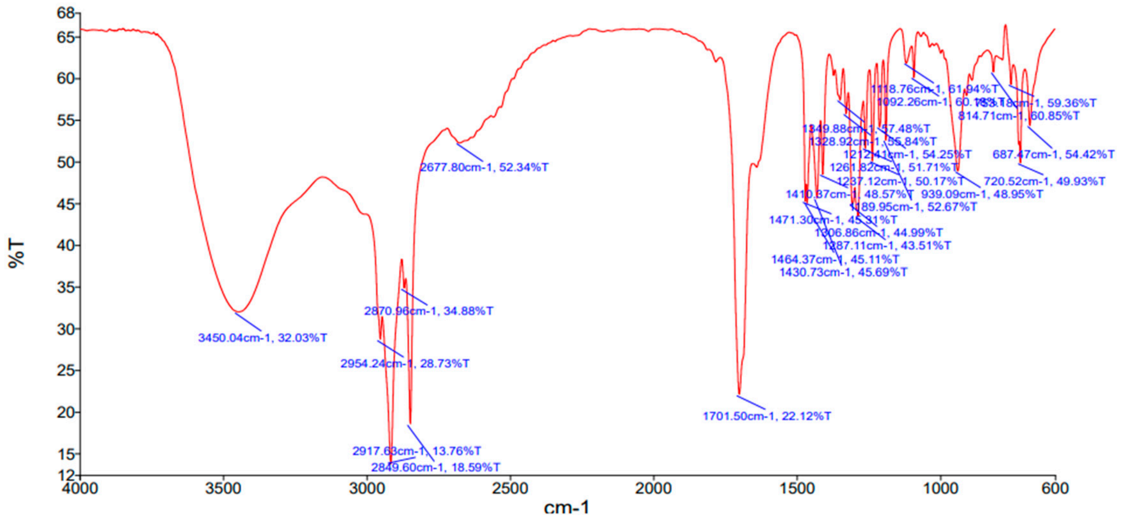

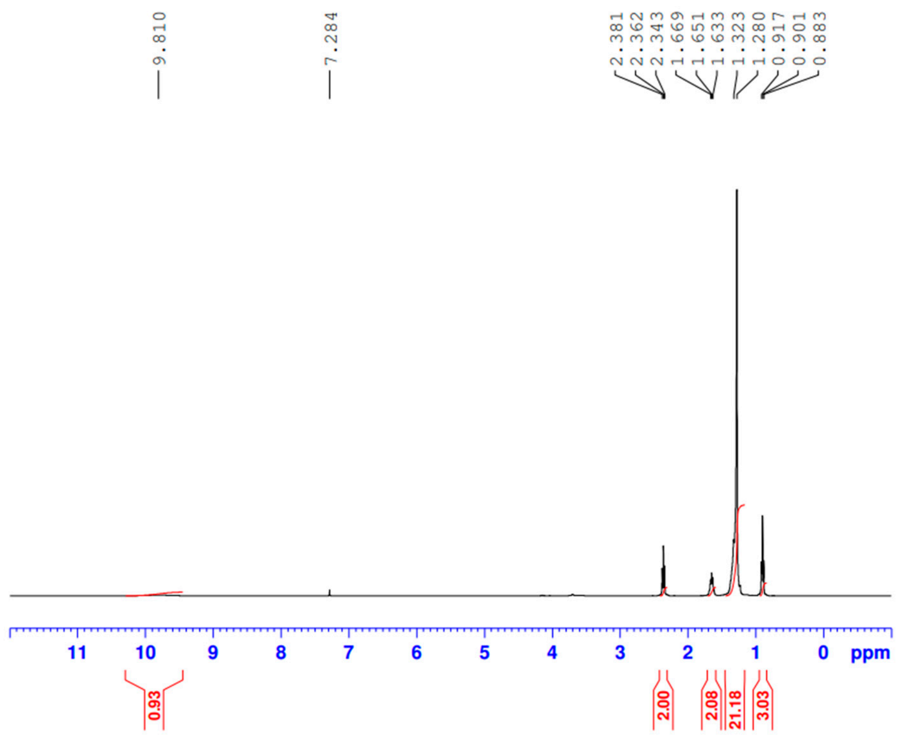
9.810
7.284
2.381
2.362
2.343
1.669
1.651
1.633
1.323
1.280
0.917
0.901
0.883
11 10 9 8 7 6 5 4 3 2 1 0 ppm
0.93
2.00
2.08
21.18
3.03

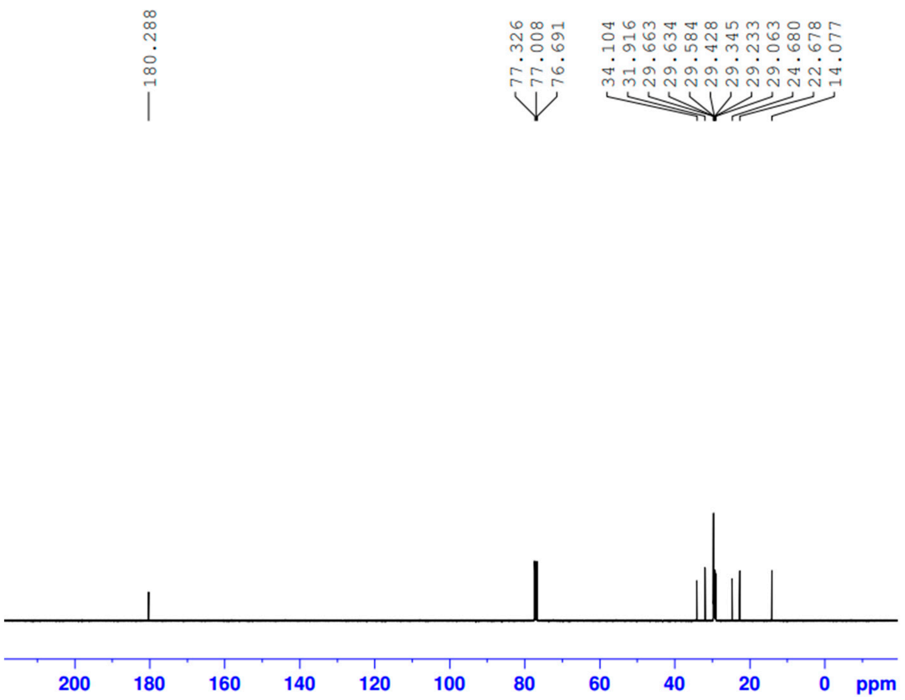
180.288
77.326
77.008
76.691
34.104
31.916
29.663
29.634
29.584
29.428
29.345
29.233
29.063
24.680
22.678
14.077
200 180 160 140 120 100 80 60 40 20 0 ppm

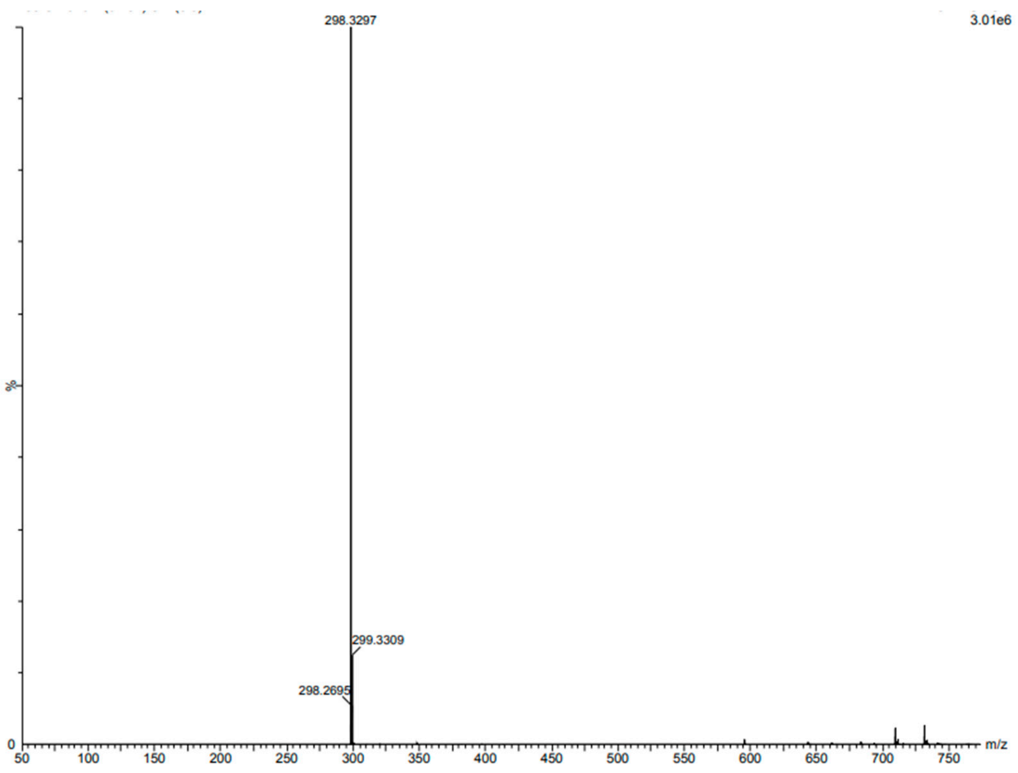

Figure S5: IR, [H]NMR, ^{13}CNMR and Mass Spectra of compound 3o

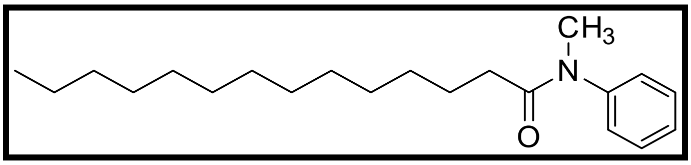

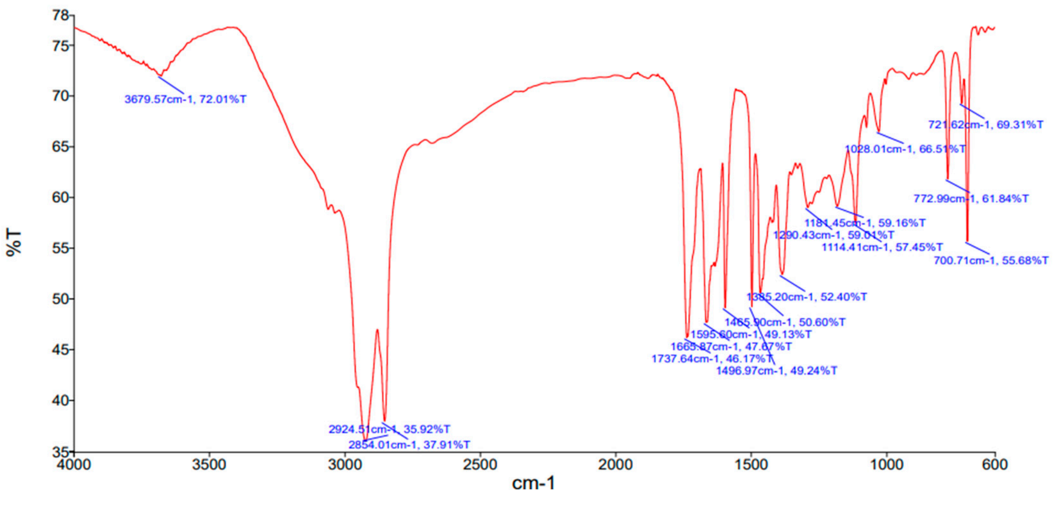

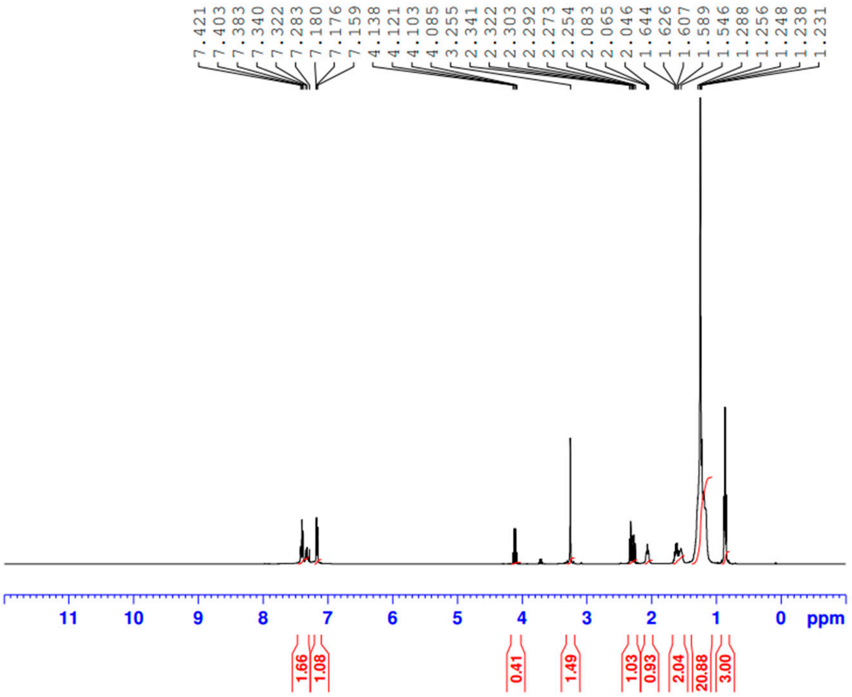
7.421 7.403 7.383 7.340 7.322 7.283 7.180 7.176 7.159 4.138 4.121 4.103 4.085 3.255 2.341 2.322 2.303 2.292 2.273 2.254 2.083 2.065 2.046 1.644 1.626 1.607 1.589 1.546 1.288 1.256 1.248 1.238 1.231
11 10 9 8 7 6 5 4 3 2 1 0 ppm
1.66 1.08 0.41 1.49 1.03 0.93 2.04 20.88 3.00

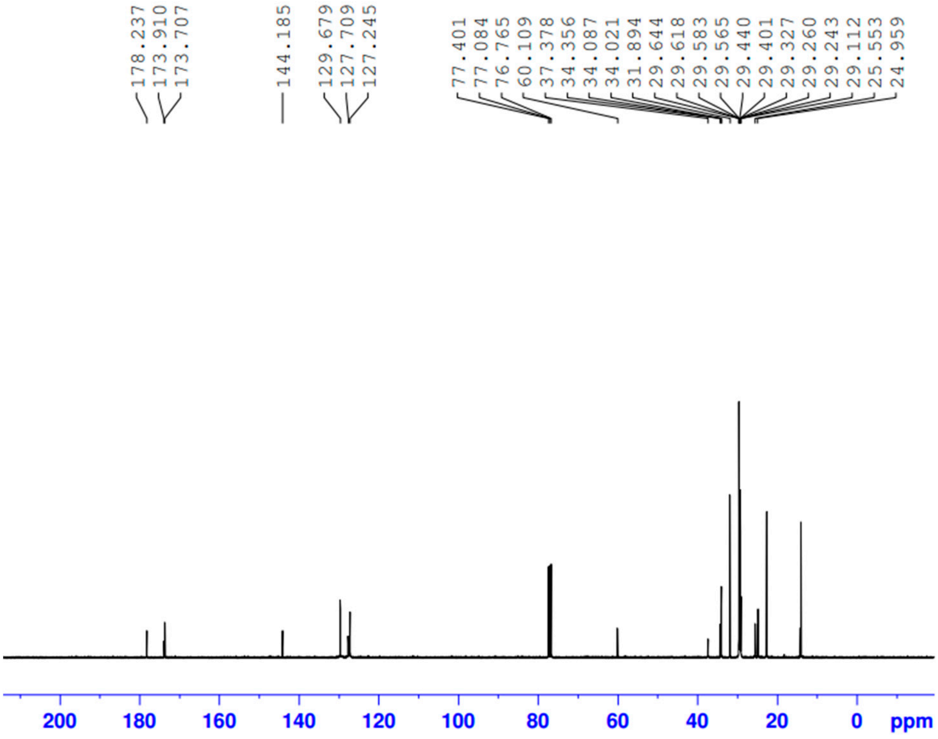
178.237 173.910 173.707 144.185 129.679 127.709 127.245 77.401 77.084 76.765 60.109 37.378 34.356 34.087 34.021 31.894 29.644 29.618 29.583 29.565 29.440 29.401 29.327 29.260 29.243 29.112 25.553 24.959
200 180 160 140 120 100 80 60 40 20 0 ppm

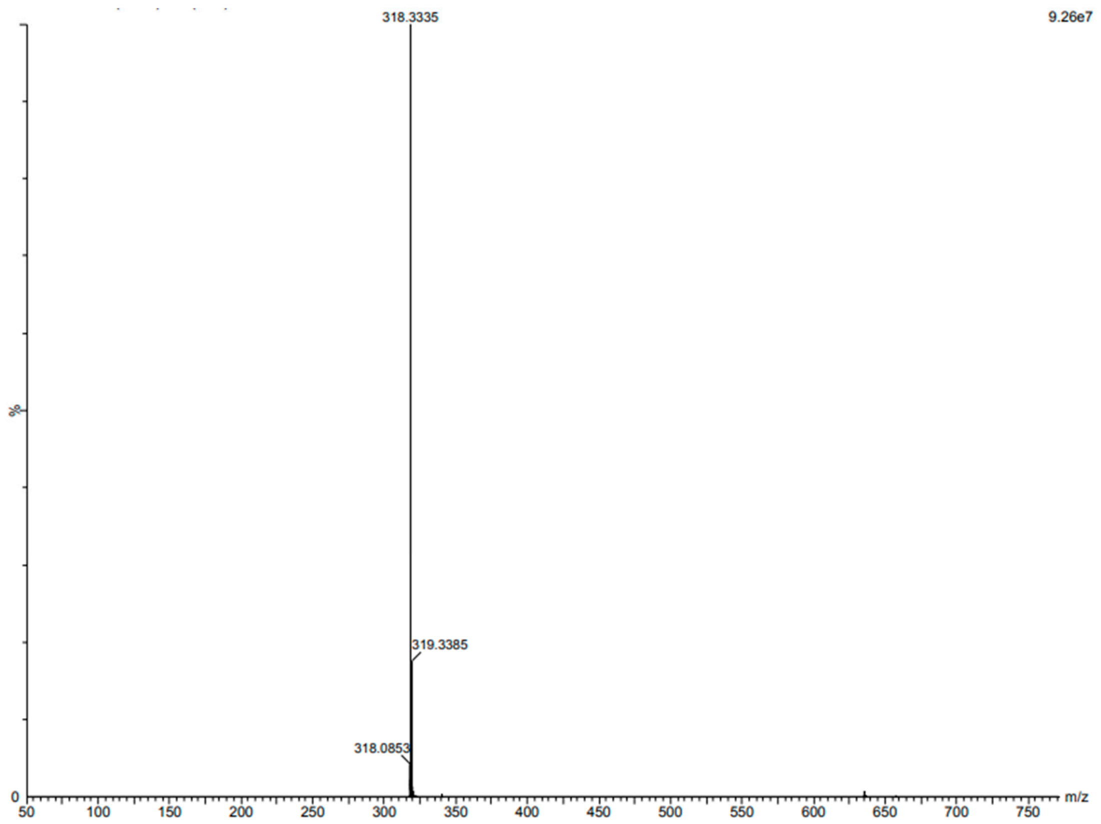

Figure S6: IR, HNMR, ^{13}CNMR and Mass Spectra of compound 3p

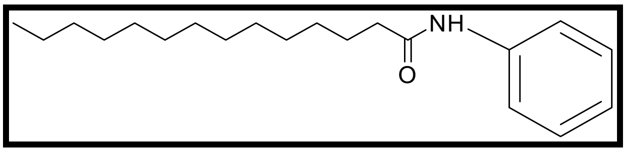

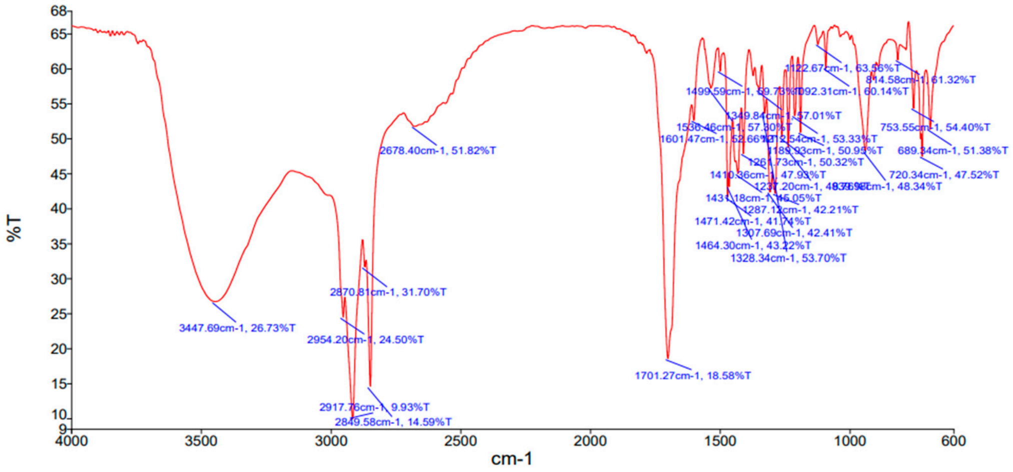

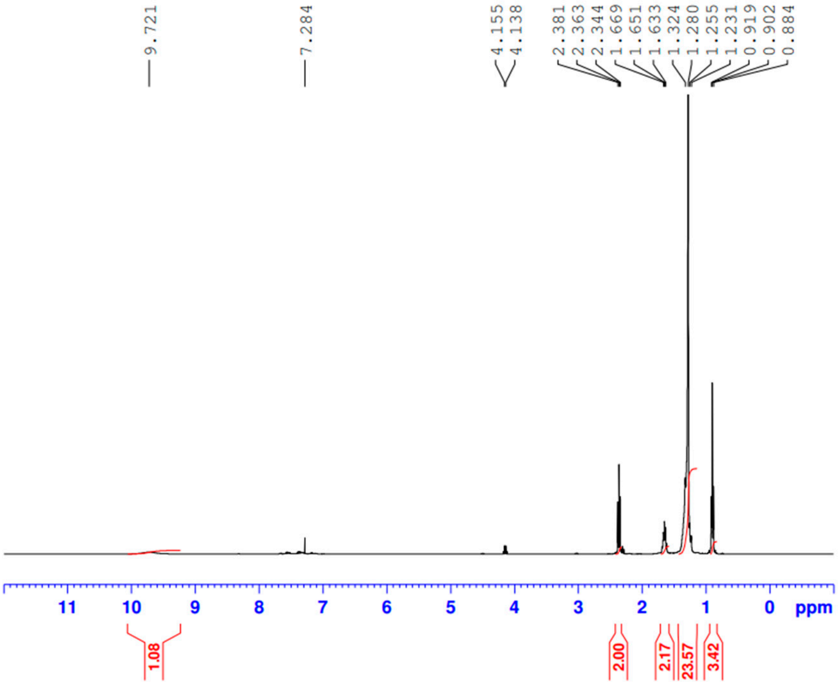
9.721
7.284
4.155
4.138
2.381
2.363
2.344
1.669
1.651
1.633
1.324
1.280
1.255
1.231
0.919
0.902
0.884
ppm
1.08
2.00
2.17
23.57
3.42

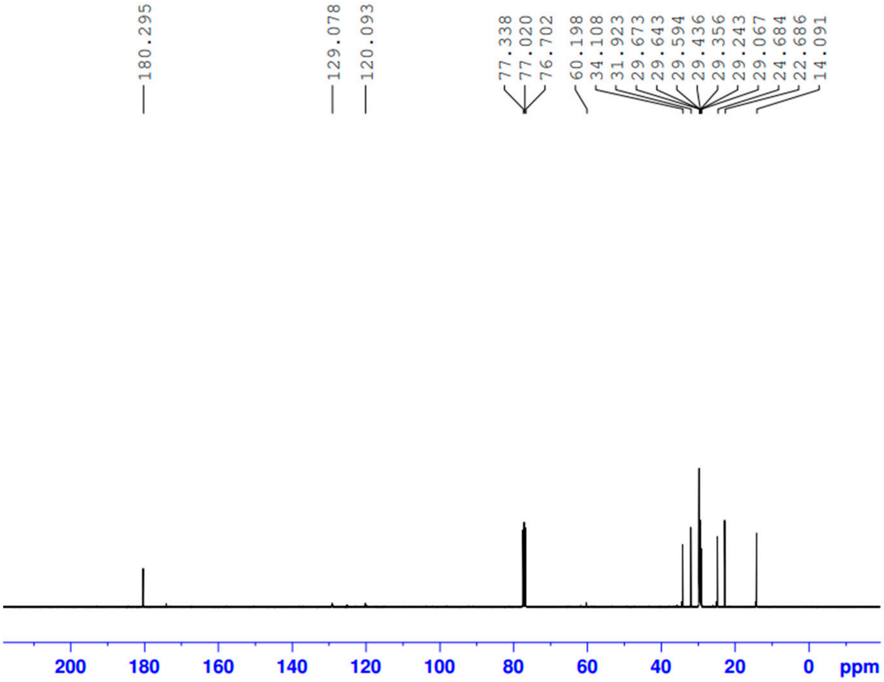
180.295
129.078
120.093
77.338
77.020
76.702
60.198
34.108
31.923
29.673
29.643
29.594
29.436
29.356
29.243
29.067
24.684
22.686
14.091
ppm

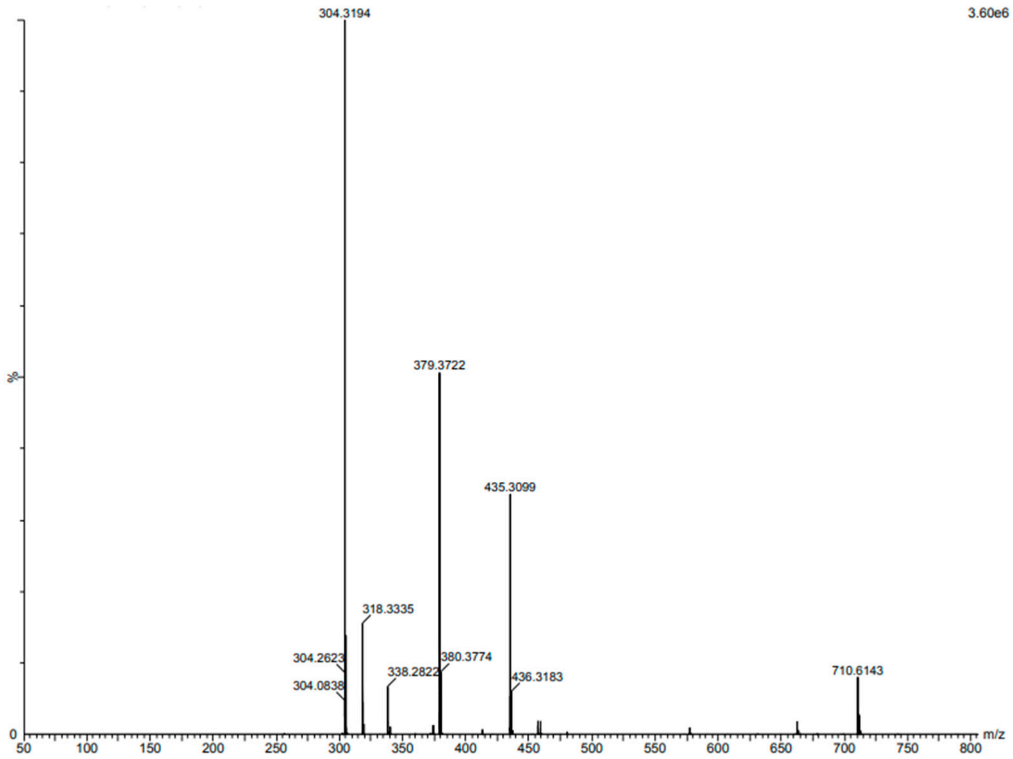
304.3194
3.60e6
%
379.3722
435.3099
318.3335
304.2623
304.0838
338.2822
380.3774
436.3183
710.6143
0
m/z
50
100
150
200
250
300
350
400
450
500
550
600
650
700
750
800

Figure S7: IR, ^{H}NMR, ^{13}CNMR and Mass Spectra of compound 3q

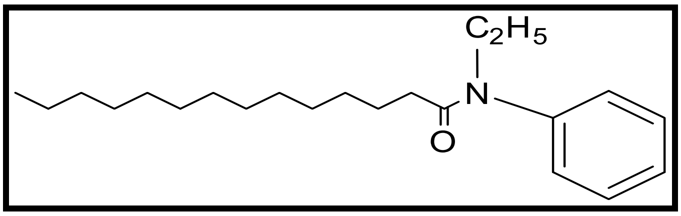

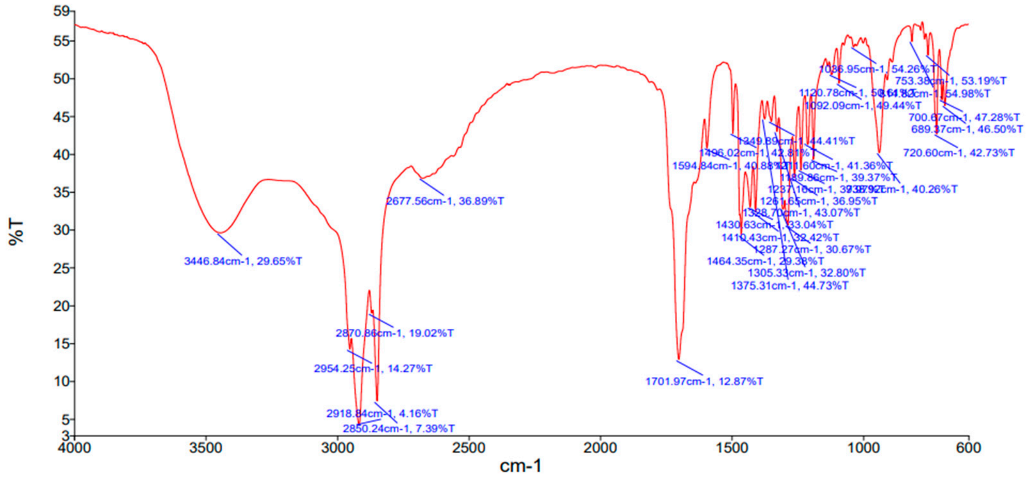

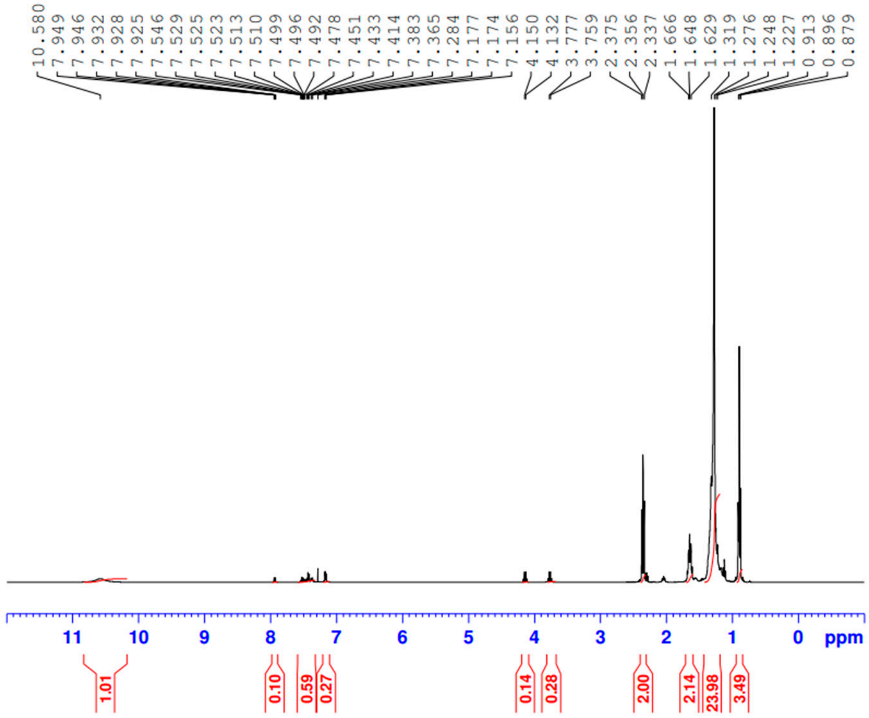

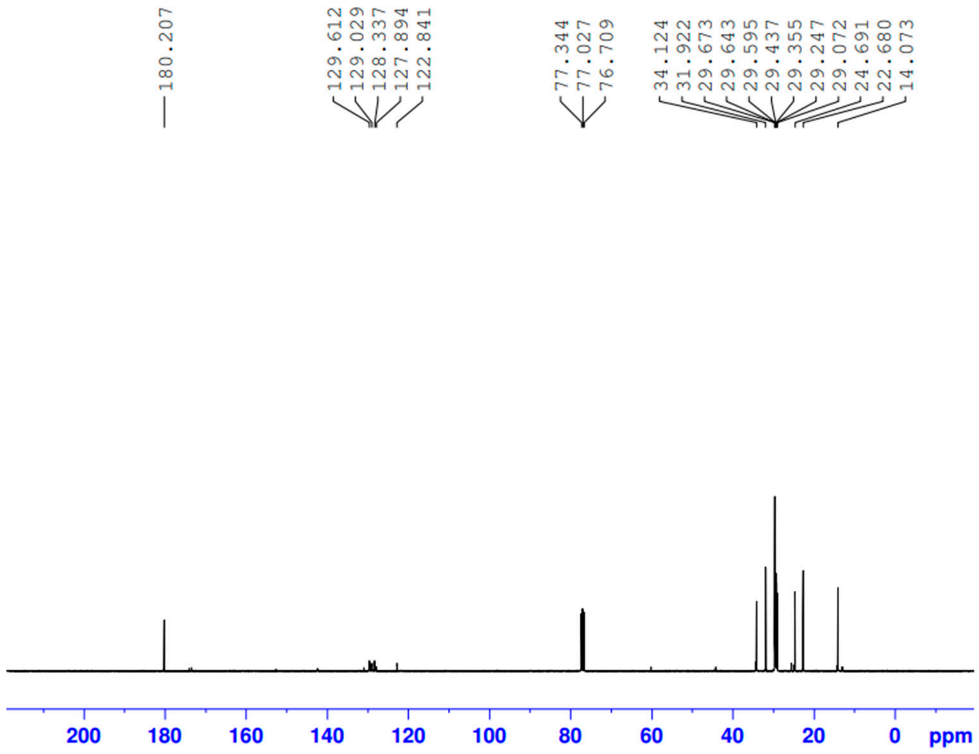
180.207
129.612
129.029
128.337
127.894
122.841
77.344
77.027
76.709
34.124
31.922
29.673
29.643
29.595
29.437
29.355
29.247
29.072
24.691
22.680
14.073
200
180
160
140
120
100
80
60
40
20
0
ppm

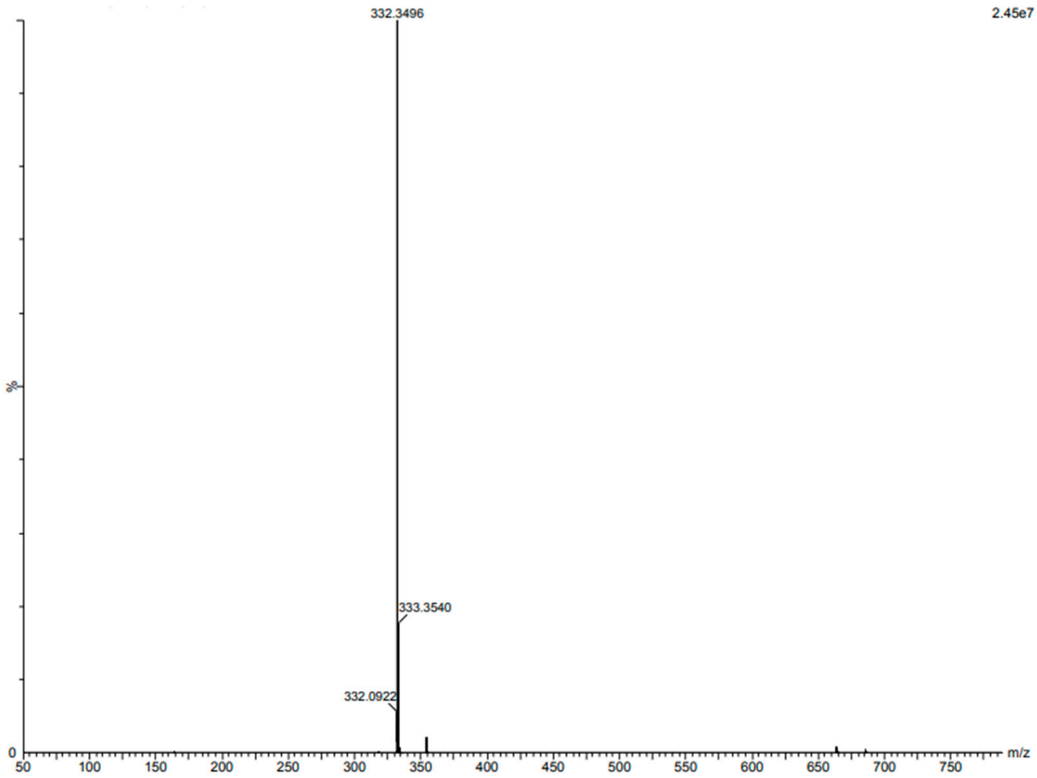
332.3496
2.45e7
%
333.3540
332.0922
0
m/z
50
100
150
200
250
300
350
400
450
500
550
600
650
700
750

Figure S8: IR, ^{H}NMR, ^{13}CNMR and Mass Spectra of compound 3r

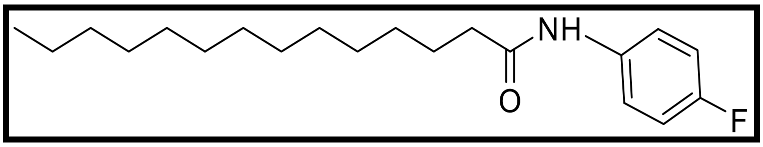

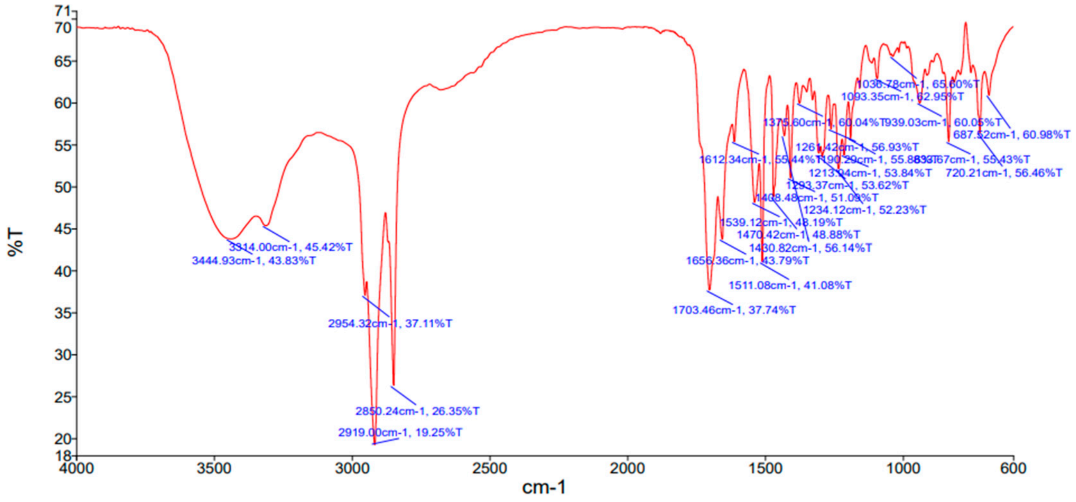

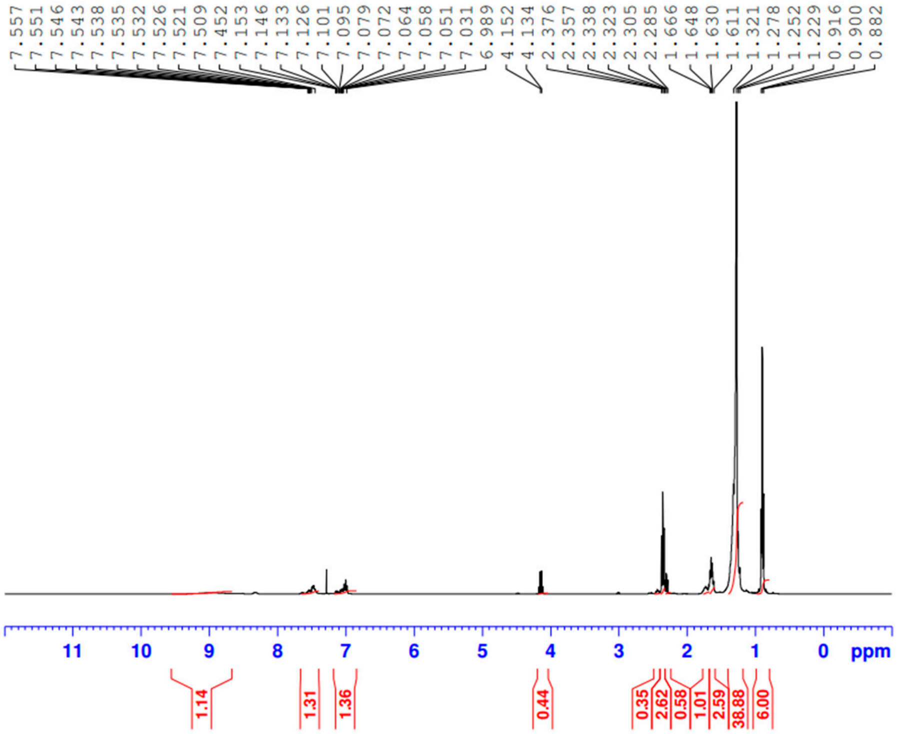

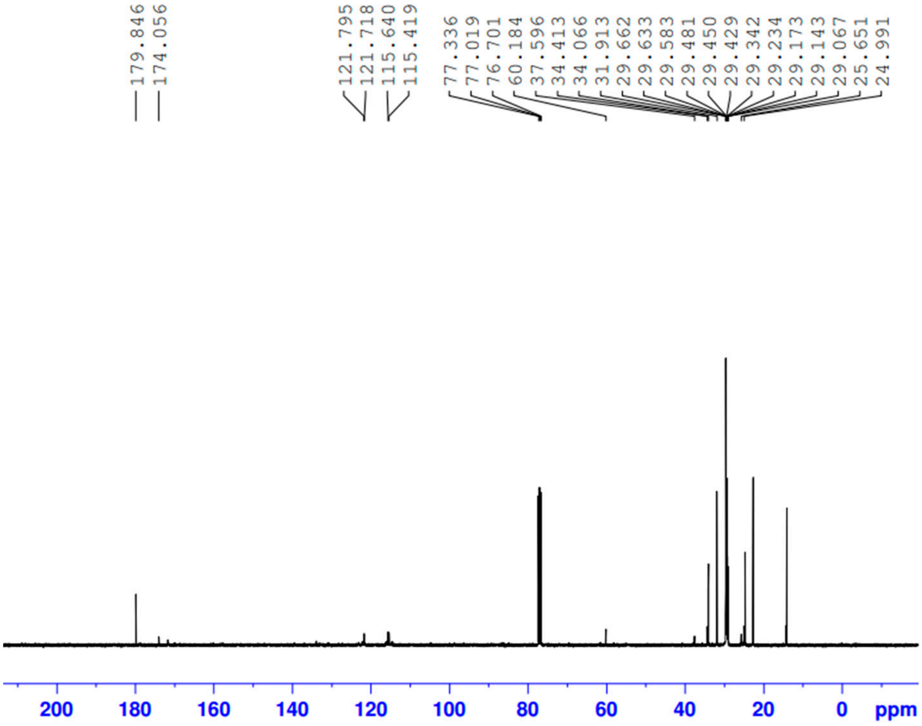
179.846
174.056
121.795
121.718
115.640
115.419
77.336
77.019
76.701
60.184
37.596
34.413
34.066
31.913
29.662
29.633
29.583
29.481
29.450
29.429
29.342
29.234
29.173
29.143
29.067
25.651
24.991
200
180
160
140
120
100
80
60
40
20
0
ppm

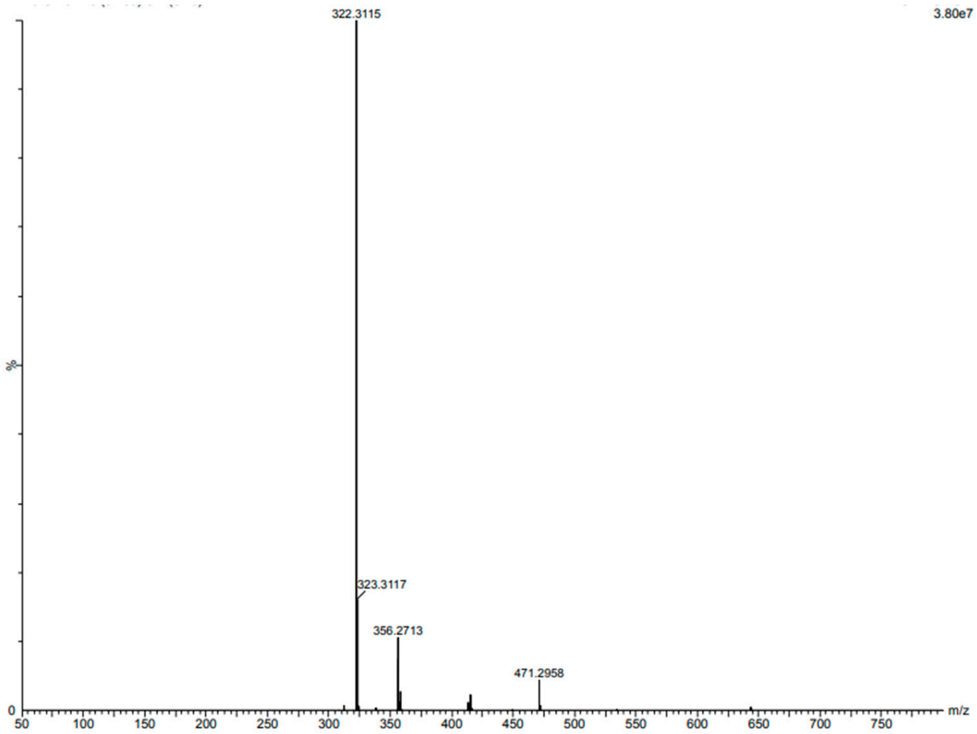
322.3115
3.80e7
%
323.3117
356.2713
471.2958
0
50
100
150
200
250
300
350
400
450
500
550
600
650
700
750
m/z

Figure S9: IR, ^{H}NMR, ^{13}CNMR and Mass Spectra of compound 3s

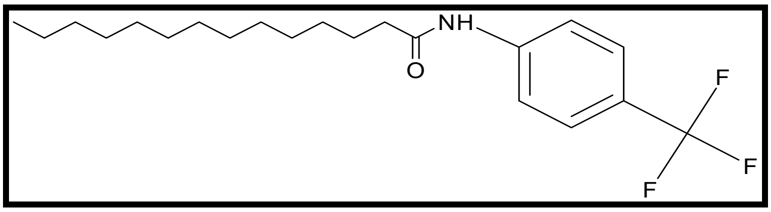

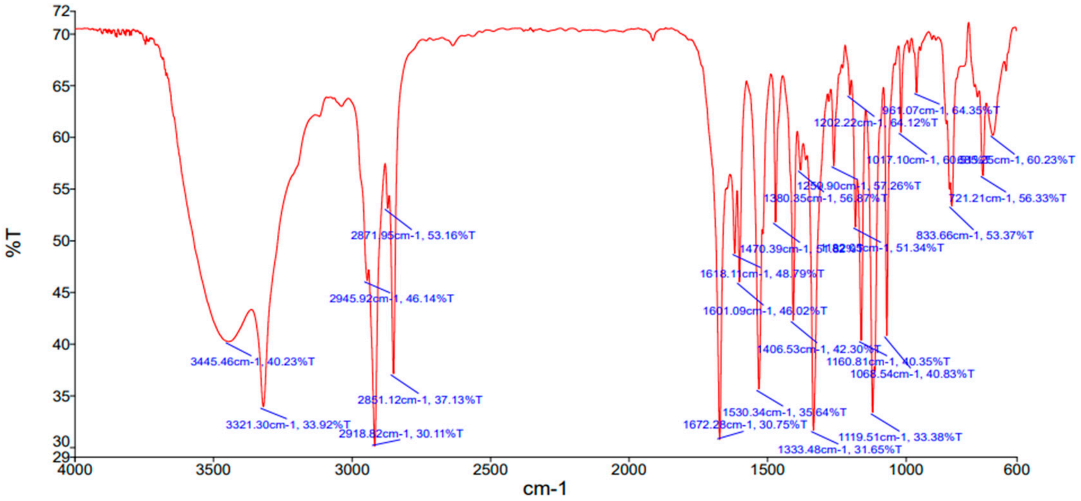

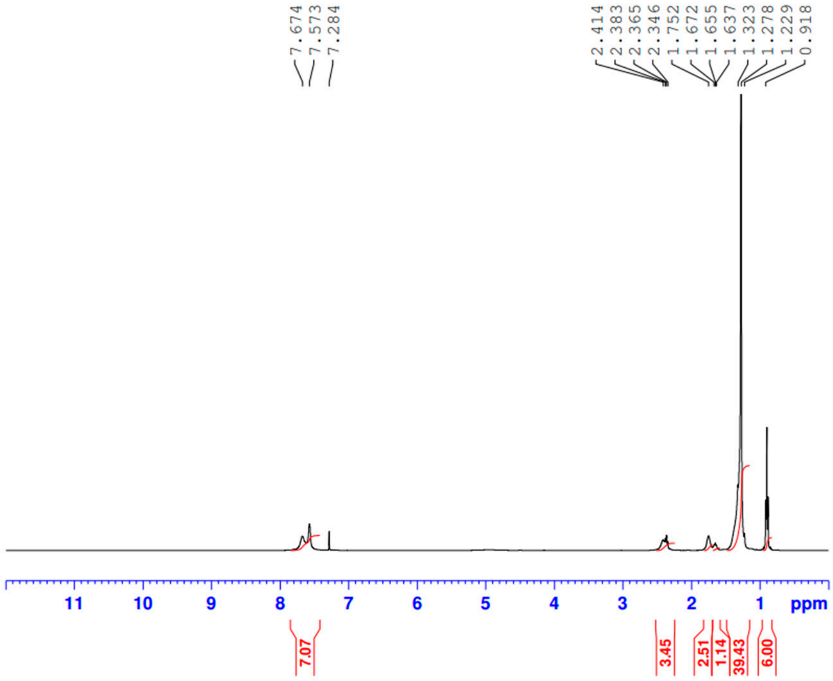

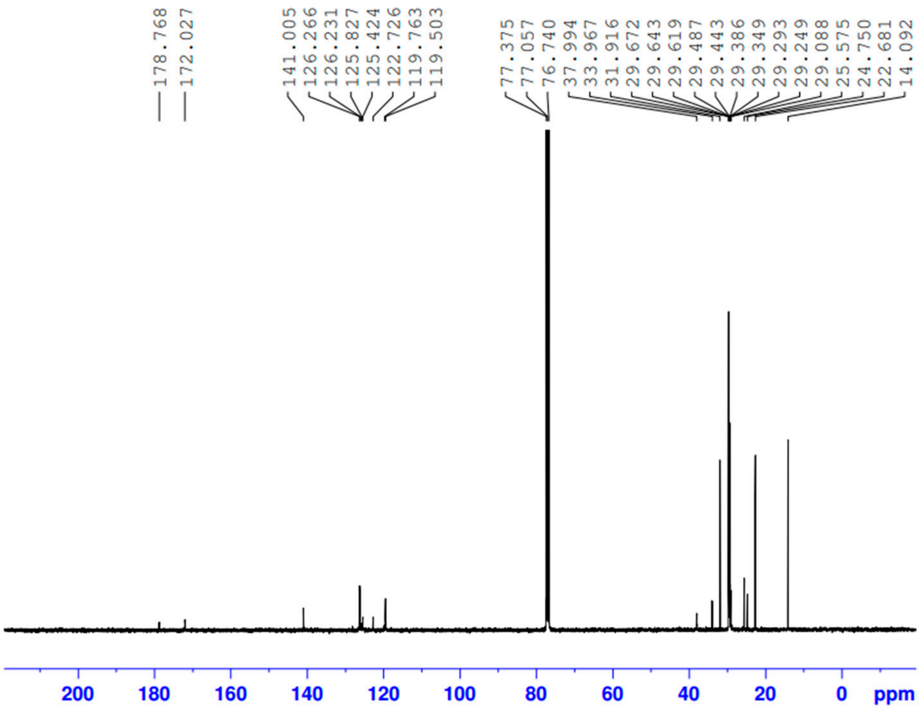

178.768
172.027
141.005
126.266
126.231
125.827
125.424
122.726
119.763
119.503
77.375
77.057
76.740
37.994
33.967
31.916
29.672
29.643
29.619
29.487
29.443
29.386
29.349
29.293
29.249
29.088
25.575
24.750
22.681
14.092
200
180
160
140
120
100
80
60
40
20
0
ppm

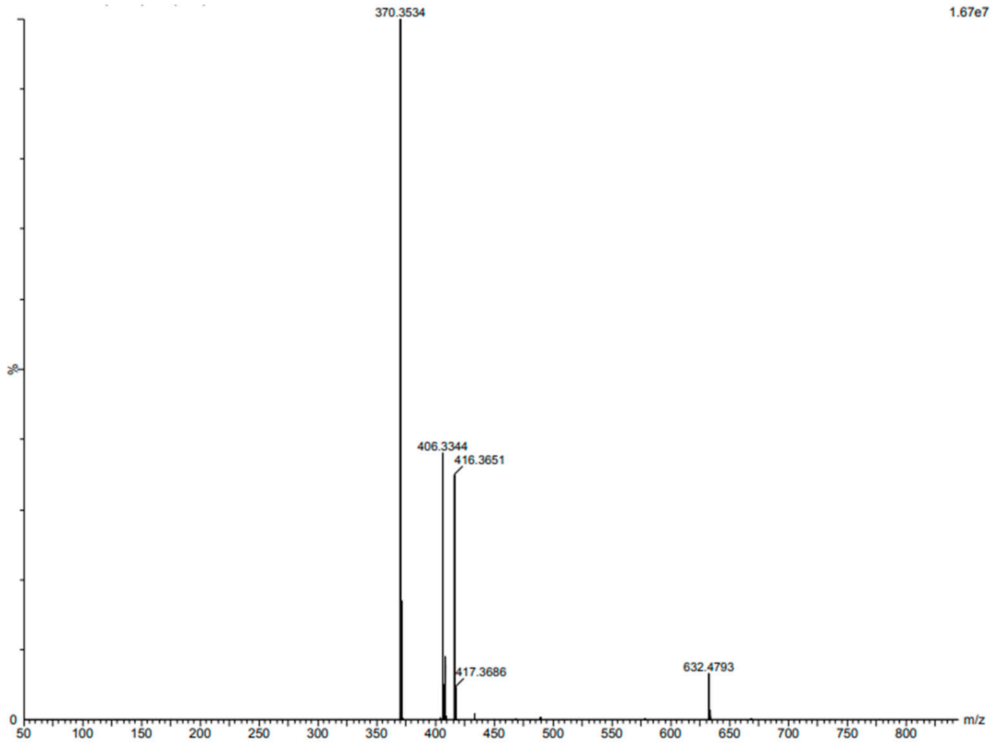

370.3534
1.67e7
%
406.3344
416.3651
417.3686
632.4793
0
50
100
150
200
250
300
350
400
450
500
550
600
650
700
750
800
m/z

Figure S10: IR, ^HNMR, ^13CNMR and Mass Spectra of compound 3t

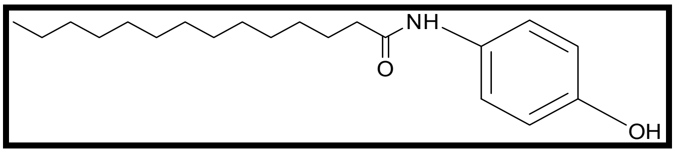

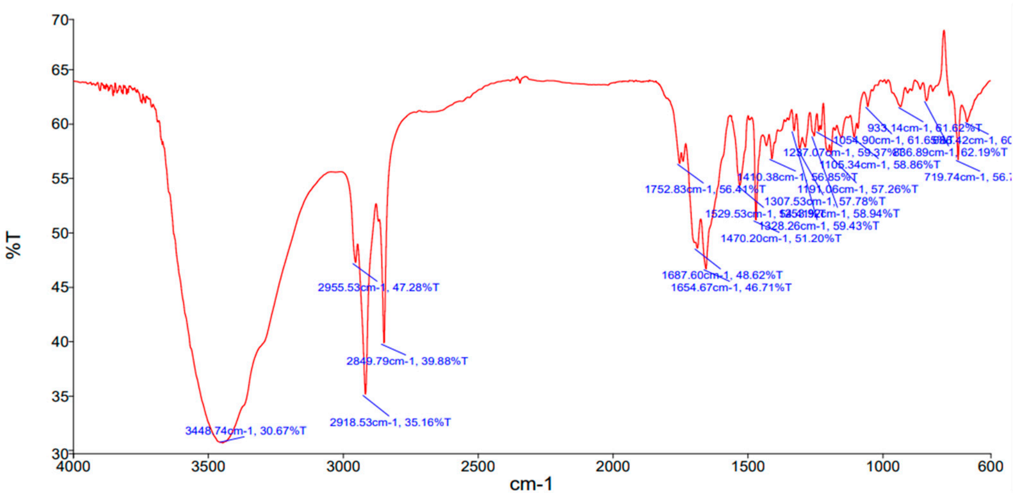

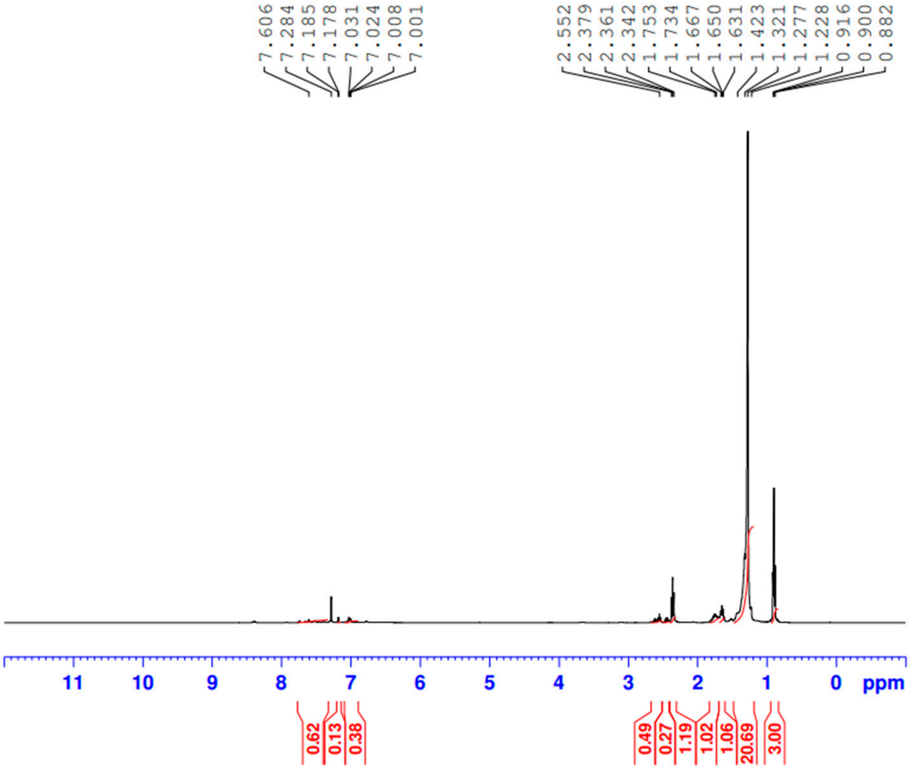

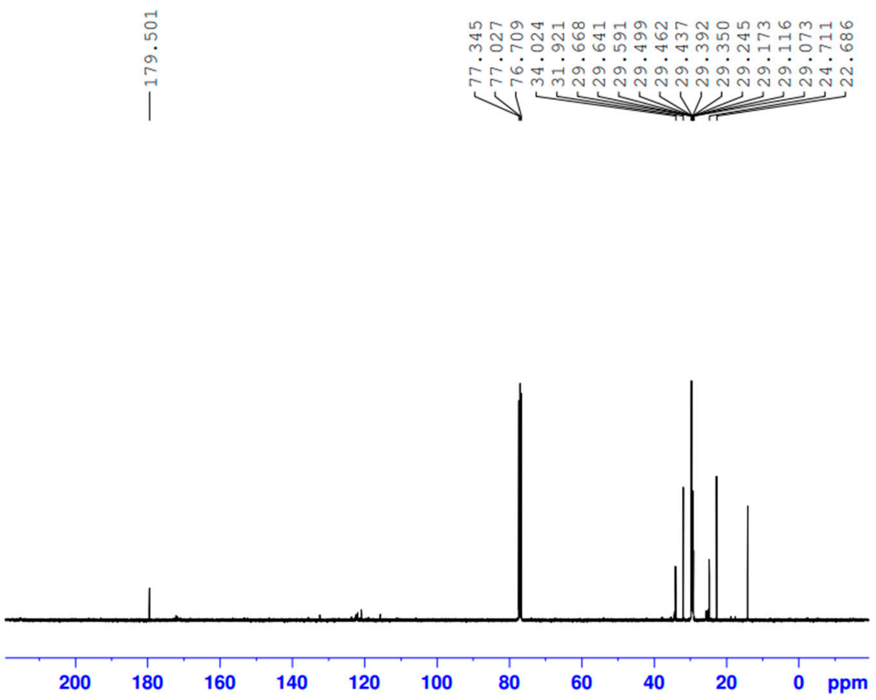

179.501
77.345
77.027
76.709
34.024
31.921
29.668
29.641
29.591
29.499
29.462
29.437
29.392
29.350
29.245
29.173
29.116
29.073
24.711
22.686
200
180
160
140
120
100
80
60
40
20
0
ppm

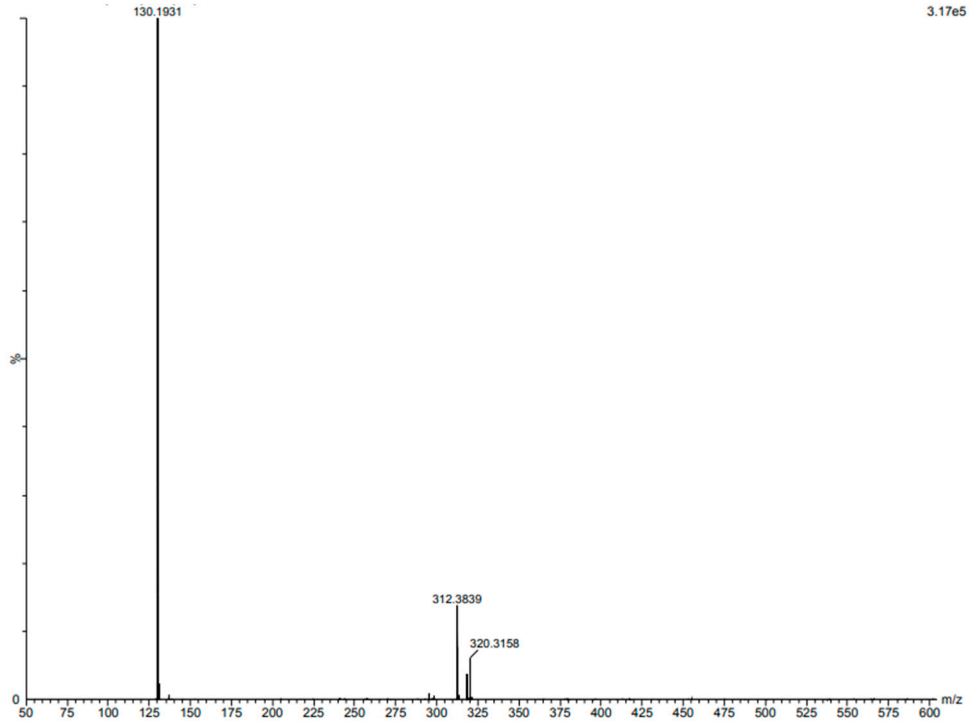

130.1931
3.17e5
%
312.3839
320.3158
m/z

Figure S11: IR, ^{H}NMR, ^{13}CNMR and Mass Spectra of compound 3u

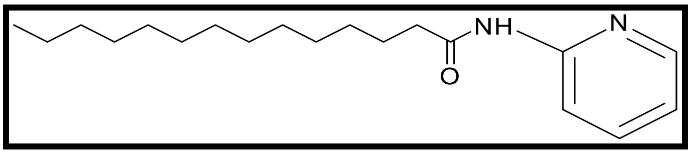

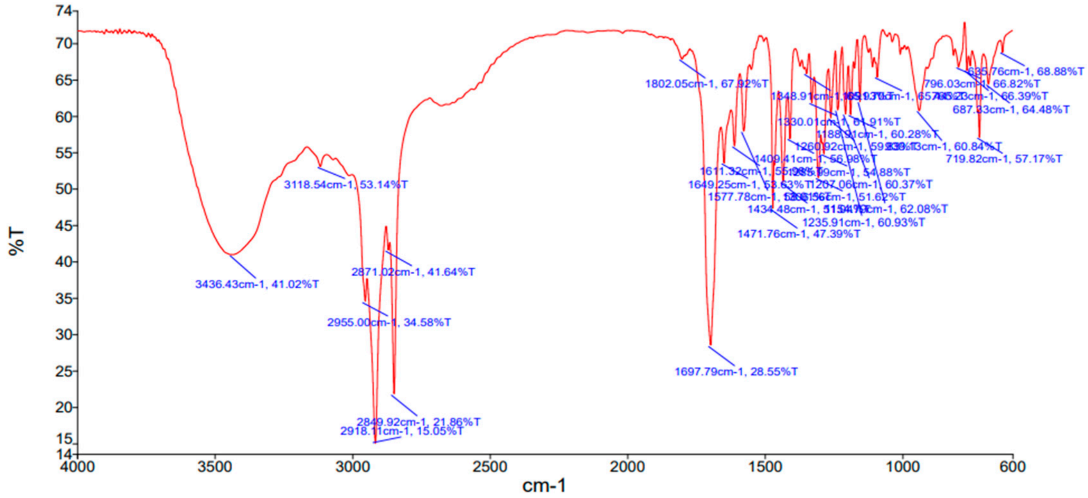

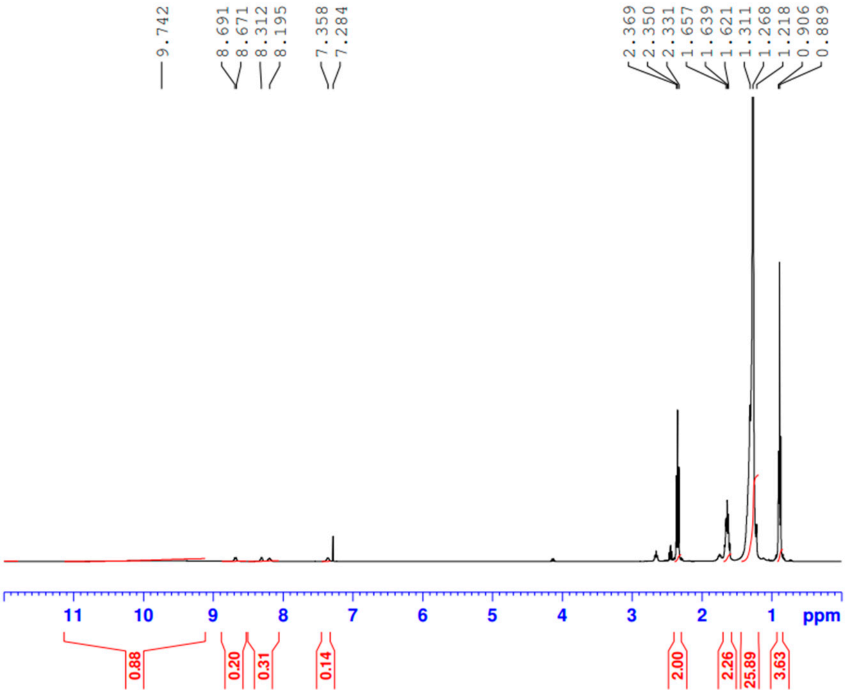

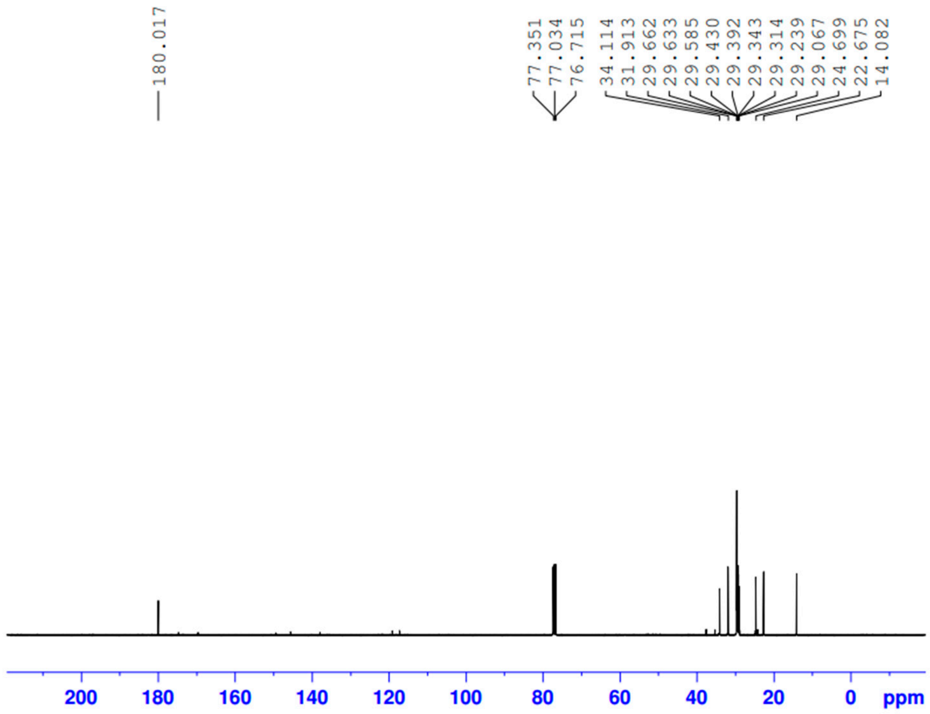
180.017
77.351
77.034
76.715
34.114
31.913
29.662
29.633
29.585
29.430
29.392
29.343
29.314
29.239
29.067
24.699
22.675
14.082
200
180
160
140
120
100
80
60
40
20
0
ppm

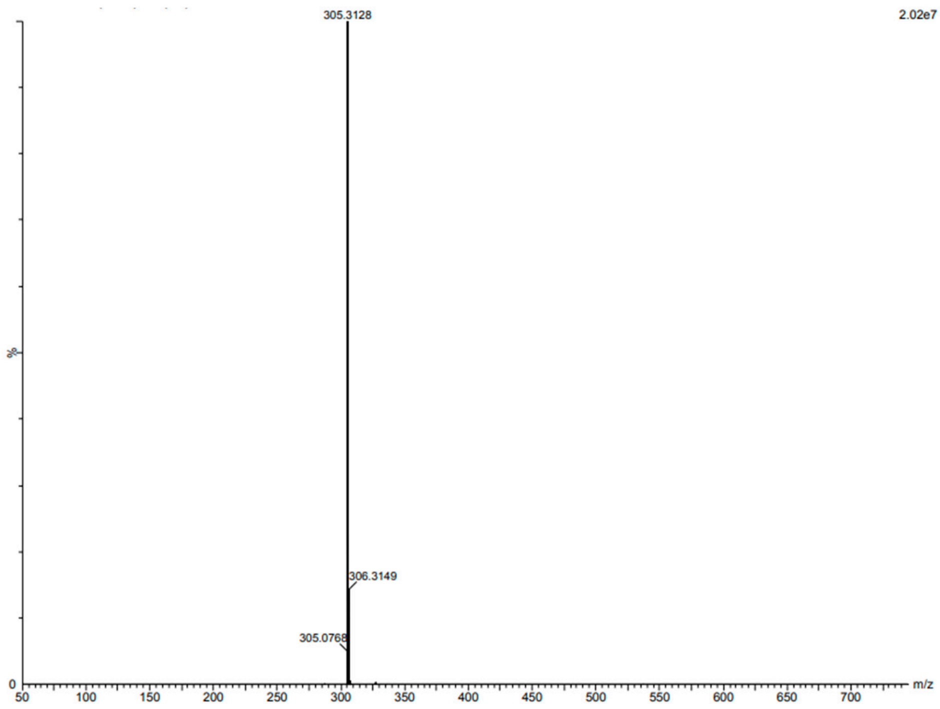
305.3128
2.02e7
%
306.3149
305.0768
0
50
100
150
200
250
300
350
400
450
500
550
600
650
700
m/z

Figure S12: IR, ^{H}NMR, ^{13}CNMR and Mass Spectra of compound 3v

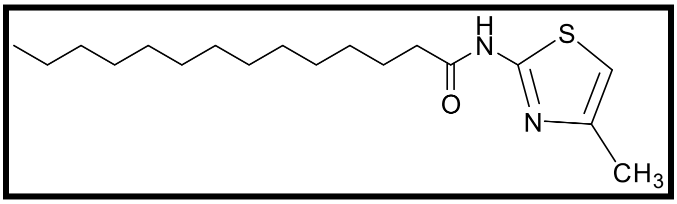

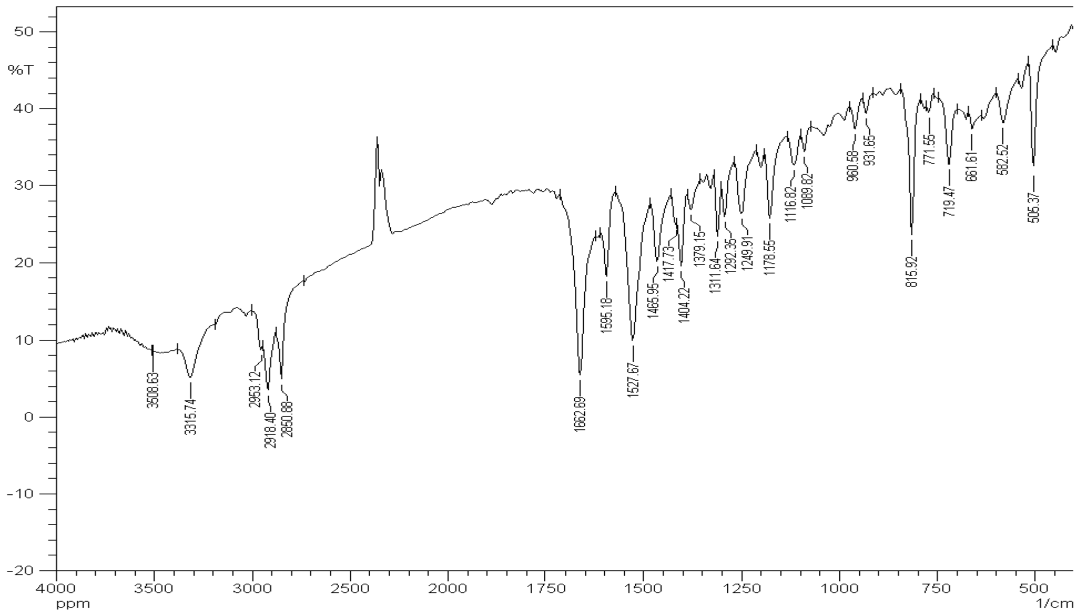

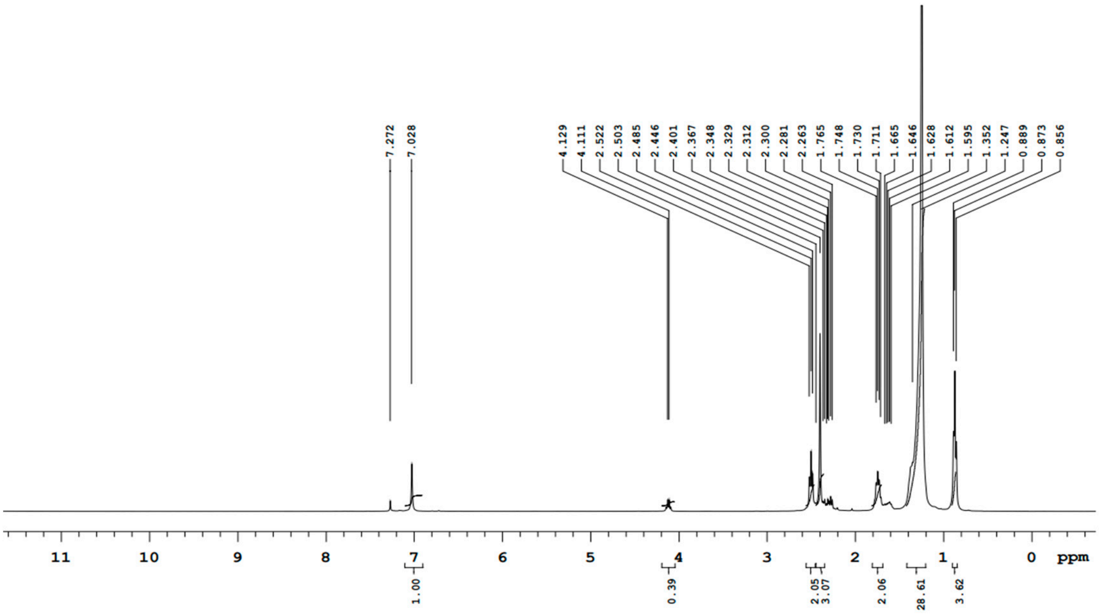

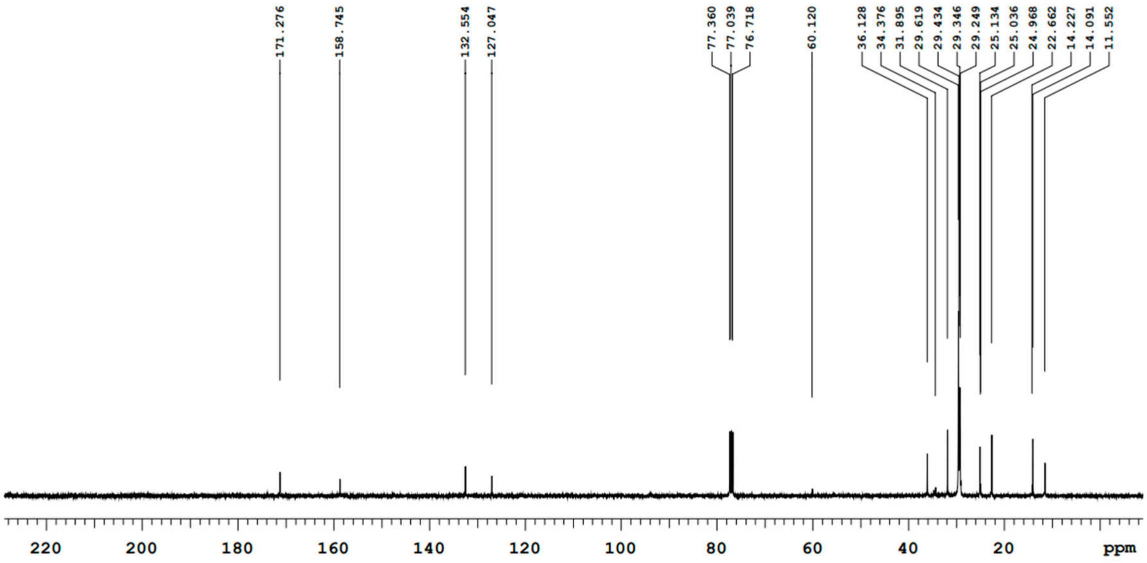
171.276
158.745
132.554
127.047
77.360
77.039
76.718
60.120
36.128
34.376
31.895
29.619
29.434
29.346
29.249
25.134
25.036
24.968
22.662
14.227
14.091
11.552
220
200
180
160
140
120
100
80
60
40
20
ppm

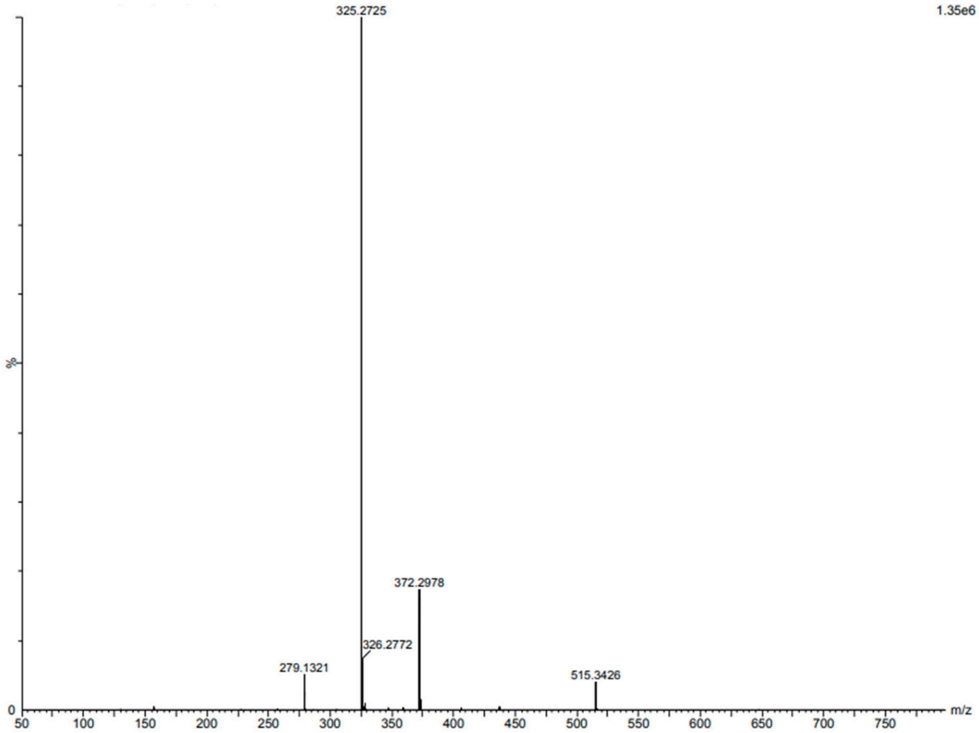
325.2725
1.35e6
%
372.2978
326.2772
279.1321
515.3426
0
50
100
150
200
250
300
350
400
450
500
550
600
650
700
750
m/z
